# Liver-specific over-expression of Cripto-1 in transgenic mice promotes hepatocyte proliferation and deregulated expression of hepatocarcinogenesis-related genes and signaling pathways

**DOI:** 10.18632/aging.203402

**Published:** 2021-09-13

**Authors:** Yu Liu, Yan-Qing Li, Shi-Hao Huang, Yong-Long Li, Jia-Wei Xia, Jun-Shuang Jia, Fang Wei, Jia-Hong Wang, Guan-Qi Dai, Yu-Cai Wang, Xiao-Yan Li, Liu-Xin Han, Xiao-Ling Zhang, Xu-Dong Xiang, Wen-Tao Zhao, Dong Xiao, Xiao-Lin Lin

**Affiliations:** 1 Cancer Research Institute, School of Basic Medical Science, Southern Medical University, Guangzhou 510515, China; 2 Institute of Comparative Medicine and Laboratory Animal Center, Southern Medical University, Guangzhou 510515, China; 3 Department of Hematology, Central Hospital of Xuhui District, Shanghai 200030, China; 4 The Third People’s Hospital of Kunming (The Sixth Affiliated Hospital of Dali University), Kunming 650041, China; 5 Department of Physiology, Faculty of Basic Medical Sciences, Guilin Medical University, Guilin 541004, China; 6 Department of Thoracic Surgery, The Third Affiliated Hospital of Kunming Medical University (Yunnan Cancer Hospital, Yunnan Cancer Center), Kunming 650118, China; 7 Department of Gastrointestinal Oncology, The Third Affiliated Hospital of Kunming Medical University (Yunnan Cancer Hospital, Yunnan Cancer Center), Kunming 650118, China

**Keywords:** Cripto-1 (CR-1), hepatocellular carcinoma (HCC), transgenic mice, hepatocarcinogenesis

## Abstract

In this study, we investigated the role of embryonic gene Cripto-1 (CR-1) in hepatocellular carcinoma (HCC) using hepatocyte-specific CR-1-overexpressing transgenic mice. The expression of truncated 1.7-kb CR-1 transcript (SF-CR-1) was significantly higher than the full-length 2.0-kb CR-1 transcript (FL-CR-1) in a majority of HCC tissues and cell lines. Moreover, CR-1 mRNA and protein levels were significantly higher in HCC tissues than adjacent normal liver tissues. Hepatocyte-specific over-expression of CR-1 in transgenic mice enhanced hepatocyte proliferation after 2/3 partial hepatectomy (2/3 PHx). CR-1 over-expression significantly increased *in vivo* xenograft tumor growth of HCC cells in nude mice and *in vitro* HCC cell proliferation, migration, and invasion. CR-1 over-expression in the transgenic mouse livers deregulated HCC-related signaling pathways such as AKT, Wnt/β-catenin, Stat3, MAPK/ERK, JNK, TGF-β and Notch, as well as expression of HCC-related genes such as *CD5L, S100A8, S100A9, Timd4, Orm2, Orm3, PDK4, DMBT1, G0S2, Plk2, Plk3, Gsta1* and *Gsta2*. However, histological signs of precancerous lesions, hepatocyte dysplasia or HCC formation were not observed in the livers of 3-, 6- or 8-month-old hepatocyte-specific CR-1-overexpressing transgenic mice. These findings demonstrate that liver-specific CR-1 overexpression in transgenic mice deregulates signaling pathways and genes associated with HCC.

## INTRODUCTION

Hepatocellular carcinoma (HCC) accounts for 70–85% of all primary liver cancer cases and is one of the most common malignant tumors in the world [1]. The 5-year recurrence rate is more than 60% and median 5-year survival rates are 35.2%, 48.3%, and 15.5% after repeat hepatectomy, ablation, and transarterial chemoembolization (TACE), respectively [2, 3]. The development of HCC involves several epigenetic and genetic changes; environmental factors such as cytotoxic and DNA-damaging chemicals, and chronic infections with hepatitis B virus (HBV) and hepatitis C virus (HCV) are common risk factors associated with HCC [4–6]. HCC is associated with aberrant activities of several signaling pathways such as Wnt/β-catenin, PI3K/AKT/GSK-3β, MAPK/ERK, TGFß/BMP, mTOR, and Stat3, as well as mutations in *p53, K-Ras, c-Myc, HBx, p16^INK4A^, APC, BRCA2, CDK2, cyclin E1, cyclin D, cyclin A* and *p38γ* genes [4–10]. However, several factors involved in the molecular pathogenesis and development of HCC are unknown and remain under extensive investigation.

Cripto-1 (CR-1) is a member of the epidermal growth factor (EGF)-Cripto-1/fibroblast growth factor related ligand (FRL1)/Criptic (EGF-CFC) protein family that coordinates primitive streak formation, mesoderm and endoderm specification, and orientation of anterior and posterior (A/P) axis during embryogenesis; it is a known marker of undifferentiated embryonic stem cells [11–16]. CR-1 is either undetected or expressed at very low levels in normal adult tissues, but is highly expressed in nasopharyngeal carcinoma and colon, lung, stomach, and breast cancers [11–16]. Aberrant activation of embryonic genes such as Oct-4 in adult tissues is frequently associated with several cancers [17–19]. Ectopic expression of the *CR-1* transgene in mammary glands induced mammary hyperplasia and adenocarcinoma in the WAP-CR-1 or MMTV-CR-1 transgenic mice [15, 20] and leiomyosarcoma of the uterus in the MMTV-CR-1 transgenic mice [21]. CR-1 plays an oncogenic role in colorectal cancer [22], melanoma [23], and esophageal squamous cell carcinoma [24].

Several studies have reported that CR-1 is associated with HCC progression [25–29]. Wang et al. demonstrated that elevated expression of CR-1 in tumor tissues was associated with high aggressiveness and poor prognosis of HCC patients [26]. Zhang et al. reported that serum CR-1 levels were significantly higher in patients with chronic hepatitis, cirrhosis, and HCC compared to healthy individuals; moreover, serum CR-1 levels were significantly higher in HBV-related HCC compared to HCV-related HCC [27]. Lo et al. reported that CR-1 positively modulated growth, tumorigenicity, invasion and chemoresistance of HCC cells by promoting stemness through stabilization of Dishevelled-3 and activation of the Wnt/β-catenin signaling pathway [25]. These findings indirectly suggested that CR-1 regulated HCC. However, direct evidence of the oncogenic role and underlying mechanisms of CR-1 in hepatocarcinogenesis are not known. Therefore, in this study, we investigated the mechanistic role of CR-1 in HCC using transgenic mice expressing hepatocyte-specific CR-1.

## RESULTS

### Truncated CR-1 mRNA is predominantly expressed in HCC cell lines and tissues

**Figure 1:**
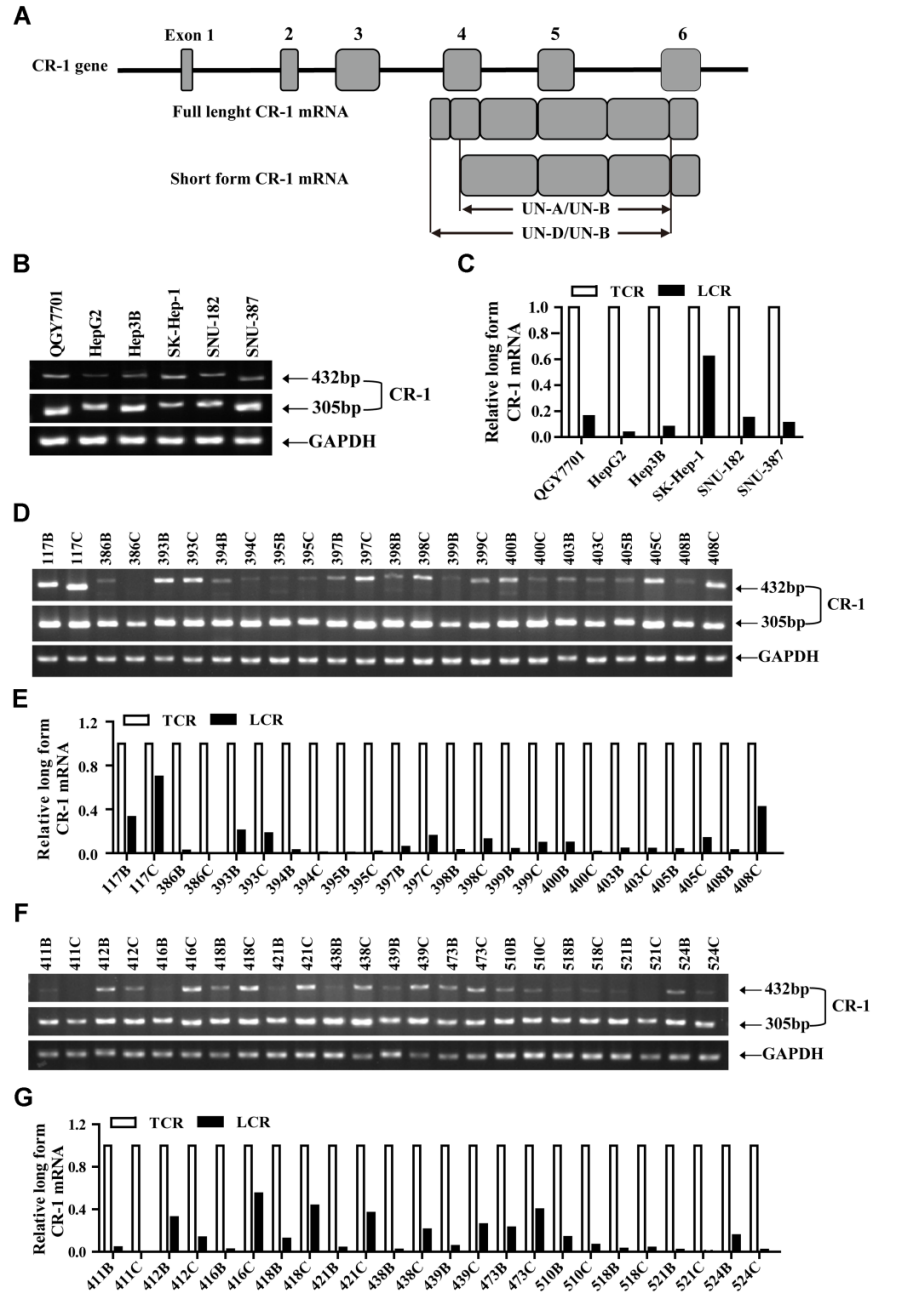
**The short form Cripto-1 (CR-1) mRNA is predominantly expressed in HCC cell lines and tissues.** (**A**) Diagrammatic representation shows structure of the *CR-1* gene and mRNAs (full-length and short-length forms). UN-D/UN-B: full-length CR-1 (FL-CR1) transcript specific primer set yields 432 bp PCR product; UN-A/UN-B: total CR-1 transcript specific primer set yields 305 bp PCR product. (**B**–**C**) RT-PCR analysis shows levels of FL-CR-1 transcript relative to total CR-1 (TCR) transcript levels in human HCC cell lines, QGY7701, Hep3B, HepG2, SK-Hep-1, SNU-387 and SNU-182. GAPDH was used as internal control. (**D**–**G**) RT-PCR analysis shows levels of FL-CR-1 transcript relative to total CR-1 transcript in human HCC (T/C) and their corresponding adjacent non-tumor liver tissues (N/B).

Previous studies reported that mammalian cells and tissues expressed both full length (FL-CR-1; 2.0 kb) and truncated (SF-CR-1; 1.7 kb) CR-1 mRNAs [30, 31]. Therefore, we performed RT-PCR analysis to quantify FL-CR-1 and SF-CR1 mRNA levels in HCC cell lines and tissues using primer sets that specifically detect FL-CR1 mRNA (UND/UNB) and total CR-1 mRNA (UNA/UNB) (Figure 1A). RT-PCR analysis showed that all six HCC cell lines (QGY7701, Hep3B, HepG2, SK-Hep-1, SNU-387 and SNU-182) expressed both FL-CR1and SF-CR1 mRNAs (Figure 1B). FL-CR-1 was highly expressed in the SK-Hep-1 cells (62%), but was lowly expressed (less than 16%) in the other HCC cell lines (QGY7701, HepG2, Hep3B, SNU-387 and SNU-182 cells) (Figure 1C). This demonstrated that SF-CR-1 was the predominant CR-1 transcript in the QGY7701, HepG2, Hep3B, SNU-182 and SNU-387 cell lines.

RT-PCR analysis also demonstrated the presence of both FL-CR1 and SF-CR1 transcripts in 24 pairs of HCC and adjacent non-tumor liver tissue biopsies (Figure 1D, 1F). Furthermore, expression levels of FL-CR-1 transcripts were significantly lower (less than 20%) in majority of the HCC tissues (*n* = 18, 75%) and adjacent non-tumor liver tissues (*n* = 22, 92%) compared to SF-CR-1 levels (Figure 1E, 1G). Collectively, these data suggested that SF-CR-1 was the predominant CR-1 transcript in majority of the HCC cell lines, HCC and adjacent non-tumor liver tissues.

### CR-1 is significantly upregulated in HCC tissues

**Figure 2:**
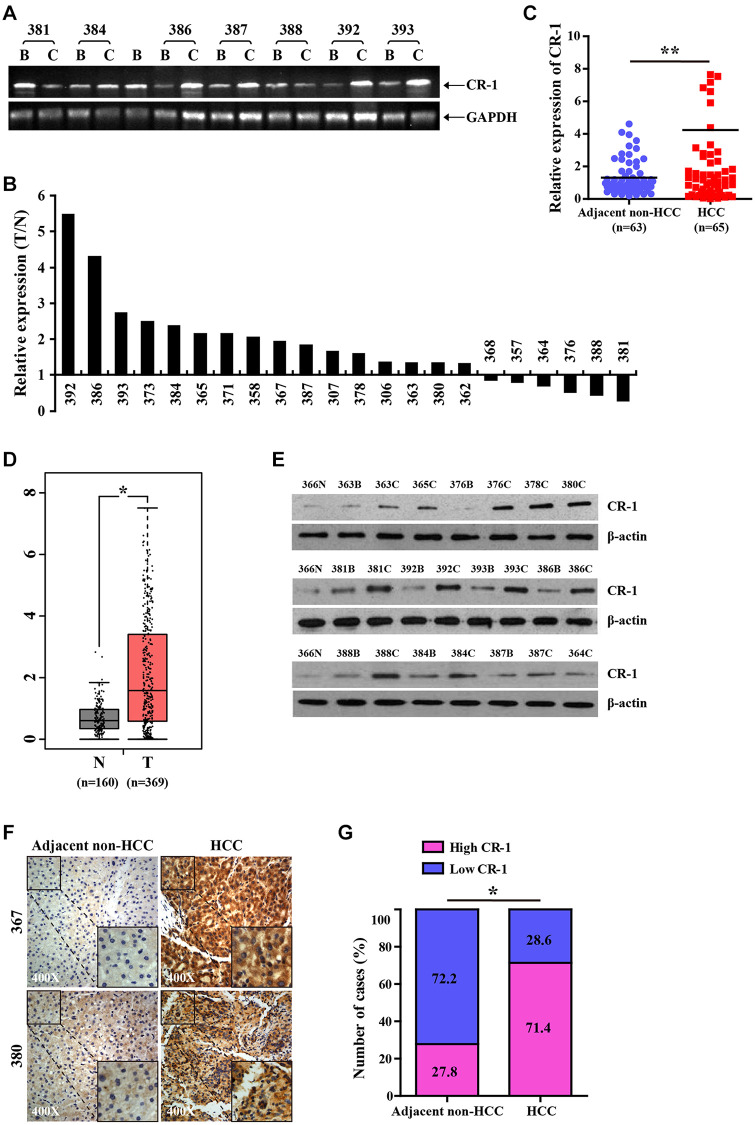
**CR-1 expression is significantly upregulated in HCC tissues.** (**A**) RT-PCR analysis of CR-1 transcript levels in HCC tissues (T) and matched adjacent non-tumor liver tissues (N). (**B**) The histogram plot shows ratio (T/N) of CR-1 mRNA levels in HCC (T) and matched non-tumor liver tissues (N) based on semi-quantitative analysis of RT-PCR data shown in Figure 2A. (**C**) qRT-PCR analysis shows relative levels of CR-1 transcript in 65 HCC (T) and 63 adjacent non-HCC liver tissue biopsies (N). (**D**) The expression levels of CR-1 transcript in HCC clinical specimens (T; *n* = 369) and non-cancerous liver tissue biopsies (N; *n* = 160) from TCGA datasets. (**E**) Representative western blot shows CR-1 protein expression levels in HCC (T) and adjacent non-HCC liver tissue biopsies (N). (**F**) Representative images show CR-1 protein expression in HCC and adjacent non-HCC liver tissue biopsies, as examined by IHC. (**G**) IHC assay analysis shows that CR-1 protein expression was significantly higher in majority of HCC tissues compared to the adjacent non-cancerous liver tissues (71.4% vs. 27.8%).

We then assessed the potential role of CR-1 in HCC progression. RT-PCR analysis demonstrated that CR-1 transcripts were significantly up-regulated in HCC tissues compared to adjacent non-tumor liver tissues (Figure 2A, 2B). Furthermore, qRT-PCR analysis confirmed that relative CR-1 mRNA expression levels were significantly higher in HCC tissues compared to the adjacent non-tumor liver tissues (Figure 2C). Moreover, CR-1 mRNA levels were up-regulated in HCC tissues from the TCGA datasets compared to the non-tumor liver tissues (Figure 2D). These results demonstrated that CR-1 expression was significantly upregulated in HCC tissues.

Western blotting analysis of 9 paired human HCC tissues, 4 unpaired human HCC tissues and 1 normal liver tissue confirmed that CR-1 protein levels were significantly upregulated in HCC tissues compared to normal liver tissues (Figure 2E). We then performed immunohistochemical staining of a tissue array with several pairs of HCC samples using a specific antibody against CR-1. The results showed that CR-1 protein levels were significantly upregulated in the HCC tissues compared to the corresponding noncancerous liver tissues (Figure 2F, 2G). These results demonstrated that CR-1 mRNA and protein levels were significantly upregulated in HCC tissues compared to the non-cancerous liver tissues.

### Generation and analysis of RCLG transgenic mice

To understand the *in vivo* functional role of CR-1 in HCC, we generated CR-1 transgenic mice (RCLG mice) (Supplementary Figure 1) using protocols as described previously [16]. We obtained two founder mice (referred to as 190^#^ and 220^#^) that strongly expressed mRFP (marker for transgenic CR-1 expression) and showed normal phenotype (Supplementary Figure 1B). In general, RCLG mice were viable and fertile, and did not manifest any gross behavioral or phenotypic abnormalities.

Next, we analyzed expression patterns of the mRFP transgene in various organs harvested from RCLG mice. MRFP expression (red fluorescence) was detected in the heart, liver, brain, lung, spleen, kidney, intestine, testis, thymus, and pancreas of the RCLG mice, but was not observed in any of the tissues from the control littermates (Supplementary Figure 2). Furthermore, mRFP expression was significantly higher in the heart, liver, kidney, lung, intestine, brain, testis, thymus, and pancreas, but was lowly expressed in the spleen (Supplementary Figure 2). Overall, mRFP was ubiquitously expressed in majority of the tissues from the RCLG mice.

Fluorescence microscopy analysis of tissue sections showed that most cells of brain, heart, kidney, lung, liver, intestine, stomach and testis from the RCLG mice were positive for mRFP expression (Supplementary Figure 3). However, some cells did not appear to be RFP positive. Therefore, we concluded that a RCLG derived mouse line was not a useful tool for investigating the role of CR-1 in HCC.

### Hepatocyte-specific overexpression of CR-1 transgene in RCLG mice mediated by Cre/*lox* P system

To further determine whether CR-1 overexpression promoted liver oncogenesis, we generated RCLG/Alb-Cre mice by crossing RCLG mice with Alb-Cre mice, in which the Cre is under the control of the hepatocyte-specific albumin (Alb) promoter [32]. The RCLG/Alb-Cre mice showed expression of Luc and CR-1 transgenes in a liver-restricted manner (Supplementary Figure 4A). Whole-animal bioluminescence imaging demonstrated Luc activity in the liver of RCLG/Alb-Cre newborn offspring, which were also mRFP-positive (Supplementary Figure 4B, 4C). This was further confirmed by PCR-based genotyping (Supplementary Figure 4D) and whole-animal bioluminescence imaging in adult mice (Supplementary Figure 4E), thereby demonstrating that Luc expression was mediated by Alb-Cre. Organ-specific bioluminescence imaging demonstrated that Luc activity was present only in the liver of Luc-positive RCLG/Alb-Cre mice (Supplementary Figure 4E) and was absent in the other organs (Supplementary Figure 4F). Luc activity was absent in all organs from the Luc-negative mouse shown in Supplementary Figure 4E (data not shown). RT-PCR and western blotting analysis showed that CR-1 mRNA and CR-1 protein levels were significantly higher in the liver tissues derived from Luc-positive RCLG/Alb-Cre mice compared to Luc-negative mice (Supplementary Figure 4G–4H). These findings confirmed liver-specific overexpression of the CR-1 transgene in the RCLG mice.

### Characterization of gross morphology and histopathology of RCLG/Alb-Cre transgenic mice

**Figure 3:**
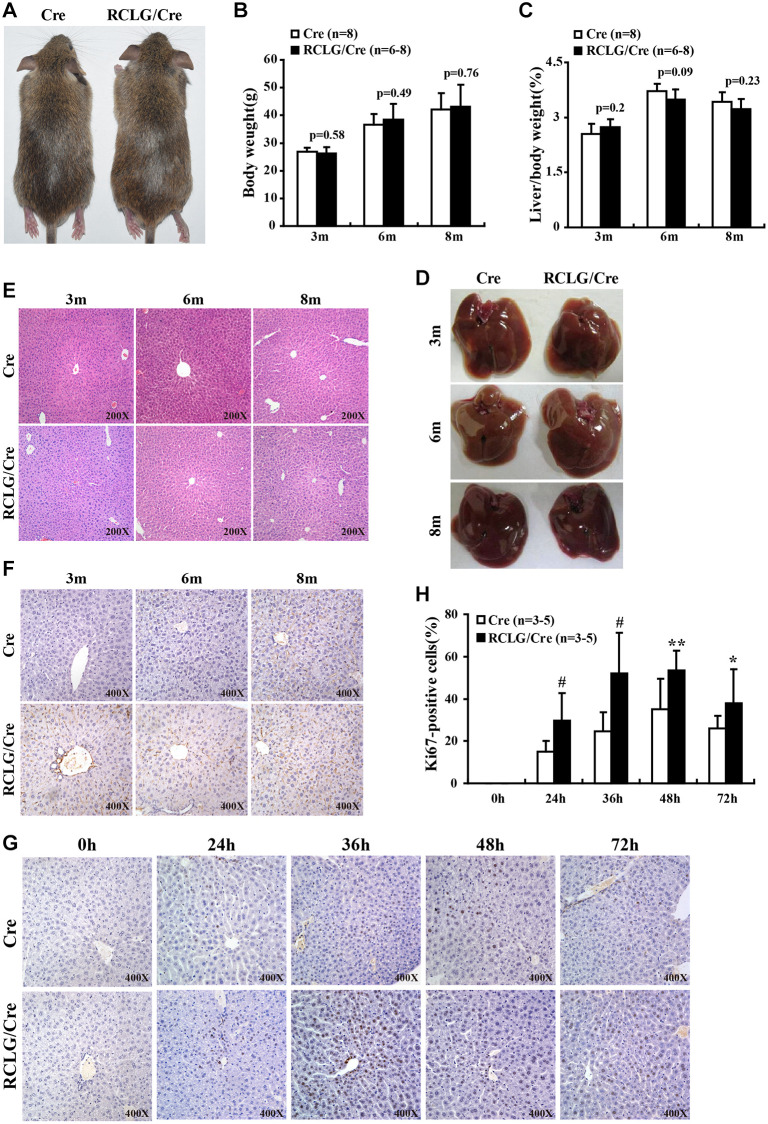
**CR-1 overexpression enhances hepatocyte proliferation in the liver after 2/3 partial hepatectomy (PHx).** (**A**) Representative images show 5-month old RCLG/Alb-Cre (right) and Alb-Cre (left) mice fed a normal diet. (**B**) Body weights of 3, 6, and 8 month-old RCLG/Alb-Cre and Alb-Cre mice. (**C**) Relative liver weights of 3, 6, and 8 month-old RCLG/Alb-Cre and Alb-Cre mice. (**D**) Representative images show gross morphology of livers from 3, 6, and 8 month-old RCLG/Alb-Cre (right) and Alb-Cre (left) mice. (**E**) Representative images of H&E-stained liver sections from 3, 6, and 8 month-old Alb-Cre and RCLG/Alb-Cre mice. (**F**) Representative Ki-67 immunostaining images show proliferation status of hepatocytes from 3, 6, and 8 month-old Alb-Cre and RCLG/Alb-Cre mice. (**G**) Representative Ki-67 immunostaining images show proliferation of hepatocytes from RCLG/Alb-Cre and Alb-Cre mice at 0, 24, 36, 48, and 72 h after PHx. (**H**) Quantitative analysis of hepatocyte proliferation based on Ki-67 immunostaining at 0, 24, 36, 48, and 72 h after PHx in RCLG/Alb-Cre (*n* = 3–5) and Alb-Cre (*n* = 3–5) mice.

Next, we analyzed the morphological and histological differences between RCLG/Alb-Cre mice and Alb-Cre control mice to determine the functional relevance of liver-specific CR-1 overexpression. The body weights (Figure 3A, 3B) and liver weights (Figure 3C) of RCLG/Alb-Cre and control mice at 3, 6 and 8 months were similar. Moreover, we did not observe any significant differences in the gross whole body morphology of mice (Figure 3A) and livers (Figure 3D) of control and RCLG/Alb-Cre mice at 3, 6 and 8 months, respectively. H&E stained liver sections from 3, 6, and 8 month old RCLG/Alb-Cre mice did not show any abnormal liver structure, increased number of mitotic hepatocytes, liver cell dysplasia or malignant liver cells (Figure 3E). We observed similar percentages of Ki67-positive nuclei in the hepatocytes from 3, 6, and 8 month old control and RCLG/Alb-Cre mice (Figure 3F). These results demonstrated that the hepatocyte-specific expression of CR-1 transgene did not cause any liver pathology such as hepatocyte hyperplasia or hepatocellular carcinogenesis.

### *In vivo* hepatocyte proliferation is increased in RCLG/Alb-Cre mice after 2/3 PHx

Next, we performed 2/3 partial hepatectomy (2/3 PHx) in RCLG/Alb-Cre mice and littermate controls to determine if hepatocyte-specific CR-1 overexpression enhanced liver regeneration. The liver tissue samples were collected on days 1, 1.5, 2 and 3 after 2/3 PHx and liver regeneration status was analyzed by determining the percentage of Ki67-positive hepatocytes. Ki67 immunohistochemistry results demonstrated that hepatocyte proliferation was significantly increased in the RCLG/Alb-Cre mice compared to their littermate controls on days 1, 1.5, 2 and 3 after 2/3 PHx (Figure 3G, 3H). These data demonstrated that CR-1 overexpression in the mouse liver enhanced hepatocyte proliferation after hepatectomy.

### CR-1 positively regulates *in vitro* HCC cell growth, migration and invasiveness

**Figure 4:**
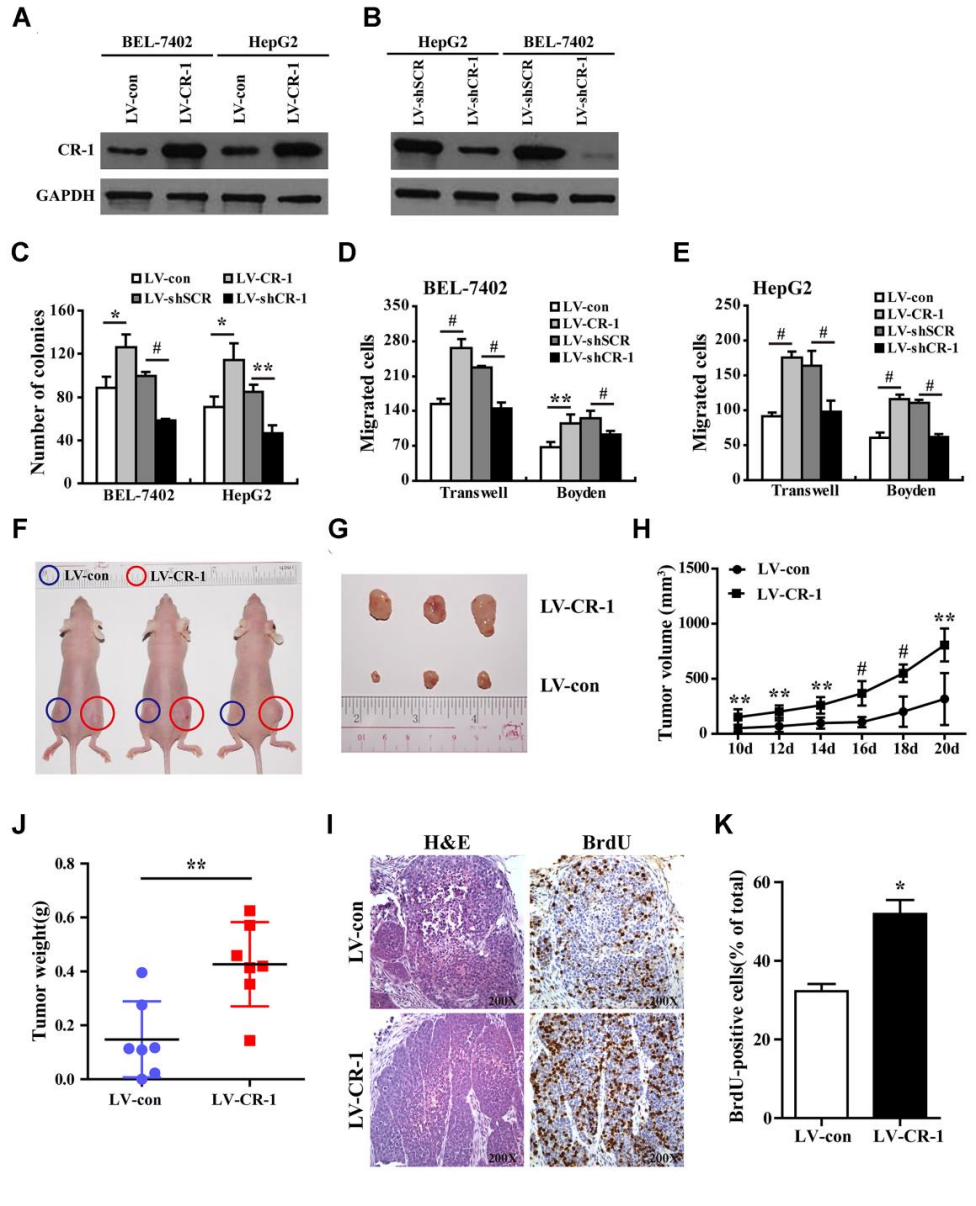
**CR-1 overexpression promotes *in vitro* proliferation, migration and invasion of HCC cells and *in vivo* xenograft HCC growth in nude mice.** (**A**) Western blot analysis shows CR-1 protein expression in control (LV-con) and CR-1-overexpressing (LV-CR-1) BEL-7402 and HepG2 cells. (**B**) Western blot analysis shows CR-1 protein expression in scrambled control (LV-shSCR) and CR-1-silenced (LV-shCR-1) BEL-7402 and HepG2 cells. (**C**) Colony formation assay results show proliferation ability of CR-1-overexpressing and CR-1-knockdown HCC cells with their corresponding controls. The representative images of this assay are shown in Supplementary Figure 5. (**D**–**E**) Transwell migration and Boyden invasion assay results show the migration and invasion abilities of CR-1-overexpressing, CR-1-knockdown, and control BEL-7402 (**D**) and HepG2 (**E**) cells, respectively. (**F**) Representative pictures of nude mice bearing subcutaneous xenografts from LV-con (blue circle) or LV-CR-1 (red circle) transduced BEL-7402 cells. (**G**) Representative images of subcutaneous xenograft tumors formed from LV-con or LV-CR-1 transduced BEL-7402 cells. (**H**) Growth curve of xenograft tumor volumes derived from LV-con or LV-CR-1 transduced BEL-7402 cells. (**I**) The weights of xenograft tumors derived from LV-con or LV-CR-1 transduced BEL-7402 cells. (**J**) Representative images of H&E-stained and BrdU-stained sections of xenograft tumors derived from LV-con or LV-CR-1 transduced BEL-7402 cells. (**K**) The percentages of BrdU-positive cancer cells in xenograft tumors derived from LV-con or LV-CR-1 transduced BEL-7402 cells, as calculated by total number of BrdU-positive cells relative to total number of cancer cells.

Since CR-1 was significantly up-regulated in HCC tissues (Figure 2), we performed loss-of-function and gain-of-function *in vitro* experiments to determine the role of CR-1 on HCC cell proliferation, migration and invasion using colony formation, transwell migration and Boyden chamber invasion assays, respectively. Western blot analysis confirmed that CR-1 was significantly overexpressed in LV-CR-1-transduced BEL-7402 and HepG2 cells compared to the corresponding controls (Figure 4A). Furthermore, western blot analysis confirmed that CR-1 protein was significantly knocked down in LV-SHCR-1-transduced BEL-7402 and HepG2 cells compared to the corresponding controls (Figure 4B). Colony formation assay results demonstrated that CR-1 overexpression significantly increased proliferation of BEL-7402 and HepG2 cells, whereas, CR-1 silencing significantly reduced proliferation of BEL-7402 and HepG2 cells (Figure 4C and Supplementary Figure 5). Transwell migration and Boyden chamber invasion assays demonstrated that CR-1 overexpression significantly increased migration and invasion of BEL-7402 and HepG2 cells, whereas, CR-1 silencing significantly reduced migration and invasion of BEL-7402 and HepG2 cells (Figure 4D, 4E and Supplementary Figure 6). These results demonstrated that CR-1 modulated *in vitro* HCC cell proliferation, migration and invasion.

### CR-1 overexpression promotes *in vivo* growth of HCC cell-derived xenograft tumors

To determine the *in vivo* role of CR-1 in HCC, we performed xenograft experiments in nude mice with subcutaneous injections of control and CR-1 overexpressing BEL-7402 cells. The subcutaneous xenograft tumors derived from CR-1 overexpressing BEL-7402 cells were significantly larger compared to those derived from control vector-transduced BEL-7402 cells (Figure 4F). The mice injected with CR-1 overexpressing BEL-7402 cells showed significantly larger tumor volumes (Figure 4H), tumor sizes (Figure 4G), and tumor weights (Figure 4I) compared to those injected with control vector-transduced BEL-7402 cells. Immunohistochemical analysis demonstrated that the percentages of BrdU-positive cells were significantly higher in tumors derived from CR-1 overexpressing BEL-7402 cells compared to those derived from control vector-transduced BEL-7402 cells (Figure 4J, 4K). Taken together, these data demonstrated that CR-1 overexpression promoted *in vivo* tumorigenicity of HCC cells.

### Molecular signaling pathways are significantly altered in the livers of RCLG/Alb-Cre mice

**Figure 5:**
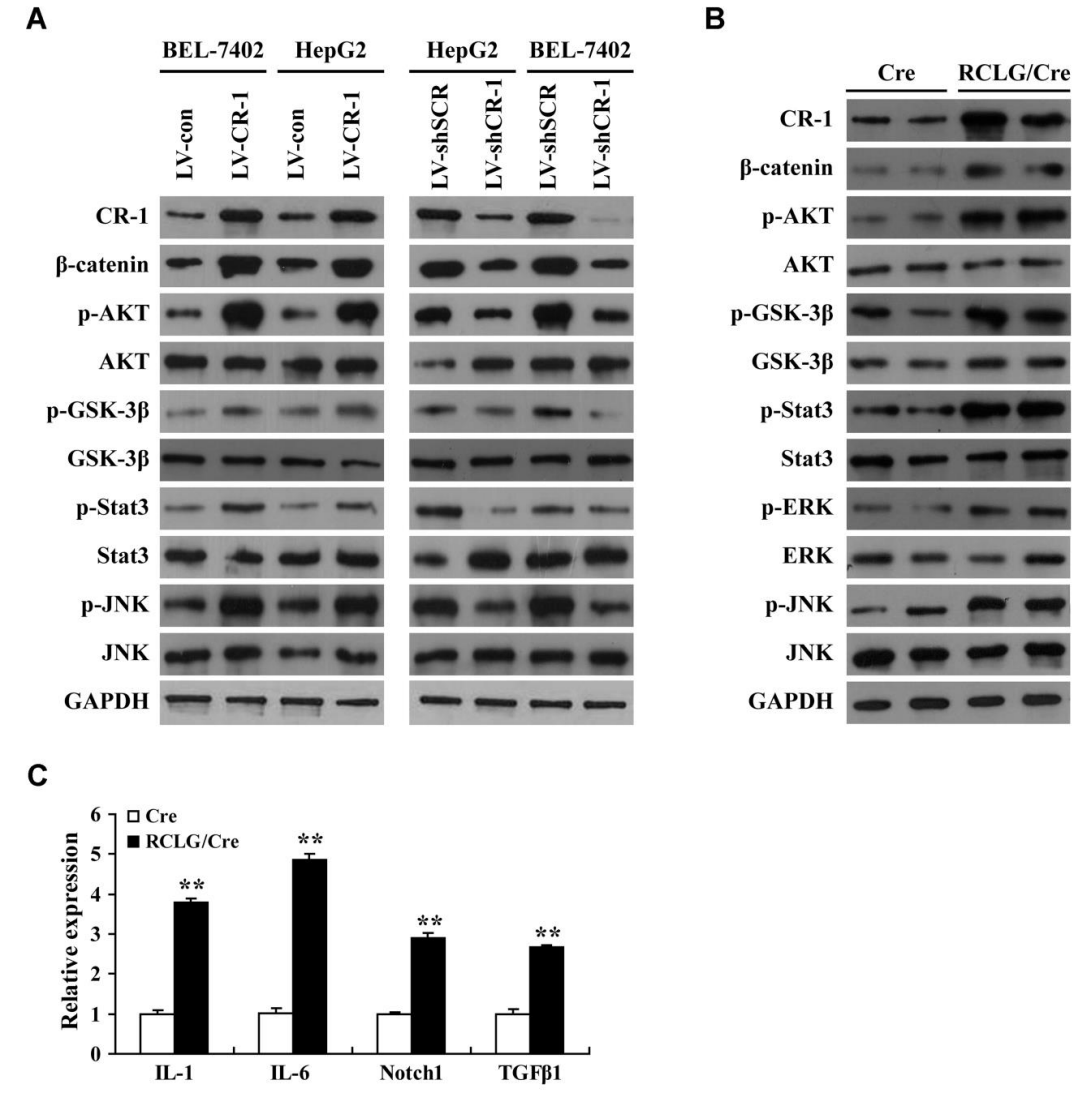
**Liver-specific overexpression of CR-1 gene in transgenic mice activates HCC-related signaling pathways.** (**A**) Representative western blot images show expression levels of the indicated proteins in CR-1-overexpressing, CR-1-knockdown, and corresponding control BEL-7402 and HepG2 cells. (**B**) Representative immunoblotting images show levels of the indicated proteins in the liver tissues of Alb-Cre and RCLG/Alb-Cre mice. (**C**) QRT-PCR analysis shows relative mRNA levels of IL-1, IL-6, Notch1, and TGF-β1 in the liver tissues of Alb-Cre and RCLG/Alb-Cre mice.

We then comprehensively analyzed the status of several molecular signaling pathways in CR-1-overexpressing and CR-1-silenced HCC cells as well as control and CR-1-overexpressing mouse hepatocytes. CR-1-overexpressing BEL-7402 and HepG2 cells showed significantly higher levels of p-AKT, p-GSK-3β, p-JNK and p-Stat3 compared to the corresponding controls (Figure 5A). This suggested that AKT, JNK and Stat3 signaling pathways were activated in CR-1-overexpressing HCC cells. Conversely, p-AKT, p-GSK-3β, p-Stat3 and p-JNK levels were significantly downregulated in CR-1-silenced BEL-7402 and HepG2 cells compared to their corresponding controls (Figure 5A). We also observed elevated levels of p-AKT, p-GSK-3β, p-Stat3, p-ERK and p-JNK in the liver tissues of RCLG/Alb-Cre transgenic mice compared to those from littermate controls (Figure 5B). β-catenin levels were significantly increased in the CR-1-overexpressing HCC cells (Figure 5A) and the liver tissues of RCLG/Alb-Cre mice (Figure 5B), but were significantly reduced in CR-1-silenced HCC cells (Figure 5A). qRT-PCR assay results demonstrated that relative levels of IL-6, IL-1, Notch1, and TGF-β1 mRNAs were significantly higher in the liver tissues of RCLG/Alb-Cre mice compared to the control mice (Figure 5C). Taken together, our results demonstrated that CR-1 overexpression in the mouse liver tissues and HCC cells significantly activated AKT, Stat3, ERK, and JNK pathways, which are closely associated with hepatocyte proliferation, liver regeneration and hepatocellular carcinogenesis.

### HCC-related genes are deregulated in RCLG/Alb-Cre livers

**Figure 6:**
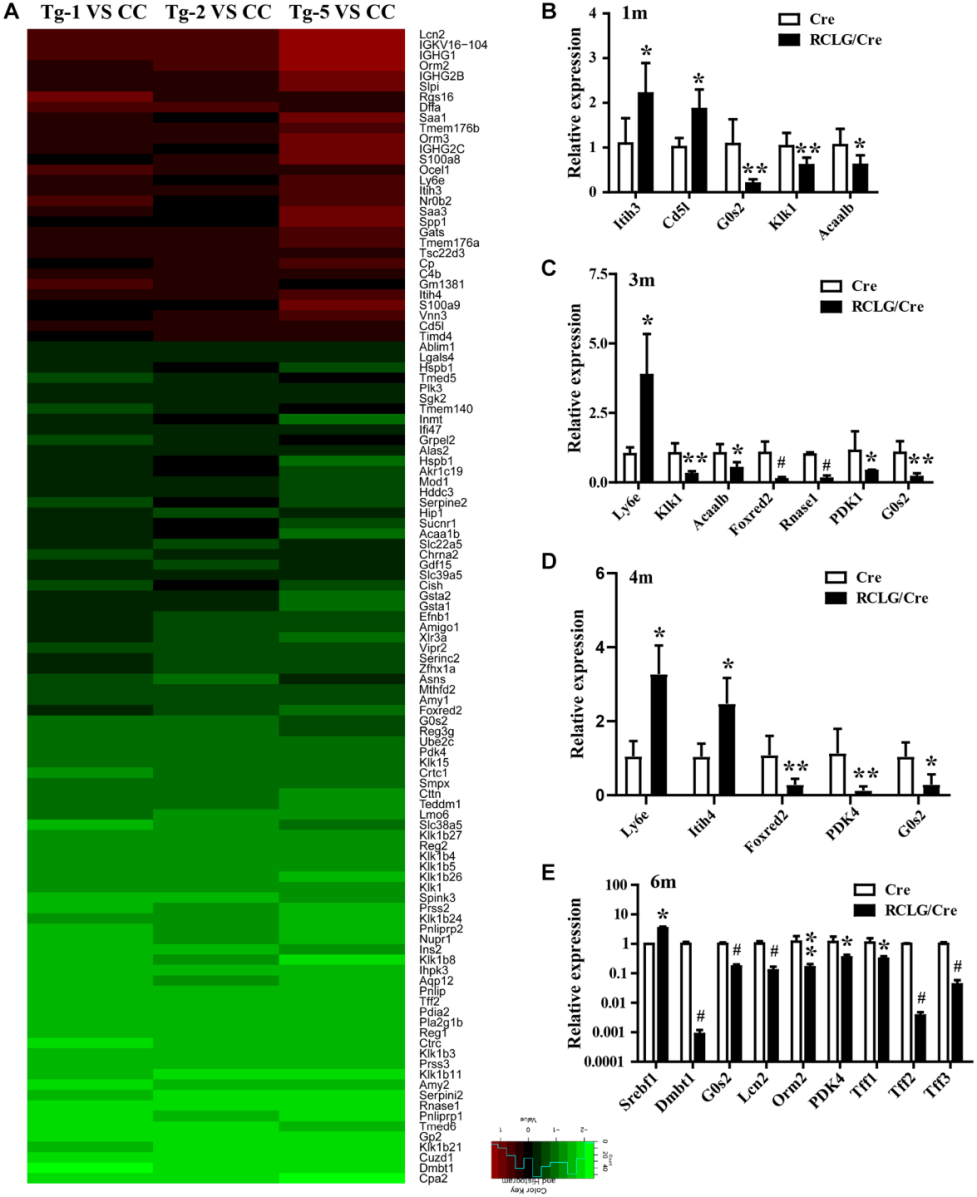
**Microarray analysis reveals altered expression of HCC-related genes in the liver of RCLG/Alb-Cre mice.** (**A**) Class comparison and hierarchical clustering of differentially expressed hepatocarcinogenesis-related genes in the livers of RCLG/Alb-Cre and Alb-Cre mice. Tg-1, Tg-2, and Tg-5 represent total RNA from the livers of three 4-month-old RCLG/Alb-Cre transgenic mice; CC represents pooled total RNA isolated from the livers of three 4-month-old Alb-Cre control littermates. Equal amounts of total RNA from the livers of each control mice were pooled to prepare CC; Tg-1, Tg-2 or Tg-5 vs. CC: Tg-1, Tg-2, or Tg-5 compared to pooled CC; Only genes showing a fold change of more than 2 and a Student’s *t* test *P* value of less than 0.05 were included in the analysis. Red indicates increased expression; blue indicates reduced expression. Other details of the microarray experiment are shown as in Supplementary Figure 7. (**B**–**E**) qRT-PCR analysis shows validation of 16 differentially expressed (increased or decreased mRNA expression) HCC-related genes from the microarray data in the RCLG/Alb-Cre and Alb-Cre mouse livers.

Despite observing altered signaling pathways that play a role in HCC growth and progression, we did not observe any precancerous lesions in the liver samples from 3-, 6- or 8-month-old RCLG/Alb-Cre mice. Therefore, we performed cDNA microarray analysis using RNA isolated from 4-month-old RCLG/Alb-Cre transgenic non-cancerous livers to determine early or pre-cancerous changes in gene expression. Microarray data analysis identified 211 genes that were differentially expressed (48 upregulated and 163 downregulated) in the non-cancerous liver tissues from the RCLG/Alb-Cre mice compared to their littermate controls (Supplementary Figure 7 and Supplementary Table 1). Among the 211 differentially expressed genes, 113 deregulated genes (upregulated: 30; downregulated: 83) (Figure 6A and Supplementary Table 2) were closely associated with cellular proliferation, apoptosis, liver regeneration, stress responses, inflammation response, immune escape, defense response, acute-phase response, cellular malignant transformation, oncogenesis or cancer malignant progression, and poor prognosis. We hypothesized that these genes were deregulated in the 4-month-old liver tissues of RCLG/Alb-Cre mice as a result of CR-1 overexpression.

qRT-PCR analysis results confirmed the expression changes of 16 genes (Itih3, Itih4, CD5L, G0S2, Klk1, Acaa1b, Ly6e, Foxred2, Rnase1, PDK4, Srebf1, Dmbt1, Orm2, Tff1, Tff2 and Tff3) in 1-, 3-, 4- or 6-month-old livers of RCLG/Alb-Cre mice (Figure 6B–6D and 6E), and were consistent with the microarray results (Figure 6A and Supplementary Table 2). These 16 genes were closely related with hepatocyte proliferation, liver regeneration and hepatocellular carcinogenesis, as reported in literature [33–54]. These findings clearly demonstrated significant changes in gene expression and activation of signaling pathways in the liver tissues of young RCLG/Alb-Cre mice, although precancerous lesions were not observed.

**Figure 7:**
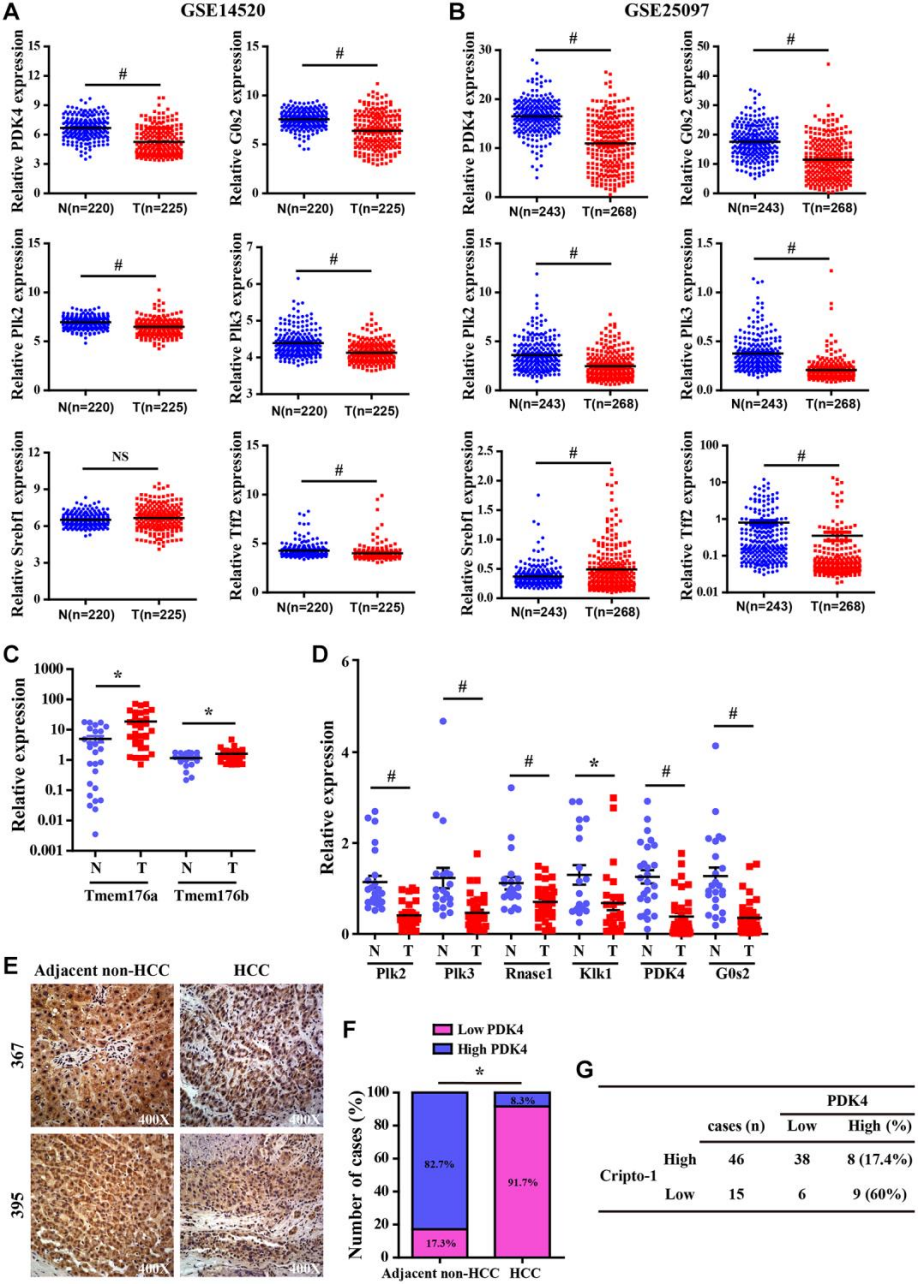
**Validation analysis of differentially expressed genes from the liver of RCLG/Alb-Cre mice in HCC tissues.** (**A**–**B**) The expression levels of CR-1 in HCC (T) and adjacent non-cancerous (N) liver tissue biopsies derived from NCBI-GEO datasets (GSE14520 and GSE25097). (**C**–**D**) qRT-PCR analysis shows expression of the indicated genes (selected from Figure 6A–6D) in primary HCC (T) and matched non-tumor liver (N) tissues. (**E**) Representative IHC images show PDK4 expression in HCC and adjacent non-tumor liver tissue biopsies. (**F**) IHC assay results show the percentage of HCC and adjacent non-tumor liver tissue biopsies with high or low PDK4 expression. As shown, PDK4 expression was significantly lower in the HCC tissue biopsies than in the adjacent non-tumor liver tissues. (**G**) The correlation analysis between CR-1 and PDK4 expression in HCC specimens based on PDK4 IHC data.

Next, we further investigated the expression levels of some differentially expressed genes (DEGs) in HCC patient datasets to determine their clinical relevance. In two GEO datasets (GEO14520 and GEO25097), the expression levels of G0S2, PDK4, Plk2, Plk3 and Tff2 in the patient HCC tissues were significantly lower than their matched adjacent normal liver tissues (Figure 7A, 7B), whereas, Srebf1 expression was upregulated in HCC tissues compared to the matched normal liver tissues (Figure 7B). These data were consistent with the results observed in RCLG/Alb-Cre transgenic mice liver tissues (Figure 6 and Supplementary Table 2). Furthermore, we selected some dysregulated genes in the RCLG/Alb-Cre transgenic livers (Figure 6 and Supplementary Table 2) and examined their expression profiles in a cohort of HCC clinical specimens by qRT-PCR. We found that the expression levels of 6 genes (PDK4, Plk2, Plk3, G0S2, Rnase1 and Klk1) were significantly reduced, and the expression levels of 2 genes (Tmem176a and Tmem176b) were significantly increased in the HCC tissues compared to the adjacent normal liver tissues (Figure 7C, 7D). Tissue array analysis showed that PDK4 levels were significantly lower in HCC specimens than in the control samples (Figure 7E, 7F). IHC analysis showed negative correlation between CR-1 and PDK4 expression levels in the HCC specimens (Figure 7G). Based on these data, we postulated that lower PDK4 levels in both RCLG/Alb-Cre transgenic livers and HCC specimens was associated with increased proliferation, tumorigenicity, motility and invasion of HCC cells. Therefore, PDK4 is a potential tumor suppressor gene for HCC in mice.

## DISCUSSION

Previous studies showed expression of both full-length (2-kb; FL-CR-1) and truncated (1.7-kb; SF-CR1) mRNA from the human *CR-1* gene in human tissues and cells [30, 31]. SF-CR-1 mRNA was only present in tissues such as the pancreas, heart, stomach, mammary gland, liver and placenta in humans [30]. Our results demonstrated that both the longer 2-kb and the shorter 1.7-kb CR-1 transcripts were expressed in the human lung, kidney, brain, testis, skeletal muscle, ovary and spleen. Baldassarre et al. showed that the shorter CR-1 transcript was predominant in majority of primary human colorectal carcinomas (7 out of 9 analyzed) and liver metastases tissues from primary colon tumors (13 out of 14 analyzed) [30]. Our results also demonstrated that the truncated 1.7-kb CR-1 transcript was predominantly expressed in the adjacent non-tumor liver and primary HCC tissues (Figure 1). Previous studies showed that NT2/D1 cells and four different human embryonal carcinoma cell lines expressed only the FL-CR-1 transcript, whereas, human colon carcinoma and hepatocarcinoma cell lines (SW480, SW620, LS174T, GEO, CBS, HepG2 and Hep3B) expressed only the truncated form of CR-1 mRNA [30, 31]. In this study, we demonstrated that both FL-CR-1 and SF-CR-1 transcripts were expressed in QGY7701, HepG2, Hep3B, SNU-182 and SNU-387 cells. However, SF-CR-1 transcript was predominantly expressed in these cells.

Baldassarre et al. reported that the full-length and short-length CR-1 mRNAs in the NT2/D1, GEO and HepG2 cell lines were not due to alterations in the genomic DNA [30]. Hamada et al. demonstrated that the expression of the SF-CR-1 transcript in HepG2 and SW620 cells was modulated by the canonical Wnt/β-catenin/TCF signaling pathway through a intronic-exonic enhancer element with three tandem TCF/LEF binding sites in the *CR-1* gene; moreover, SF-CR-1 mRNA was predominantly expressed in Wnt-active cell lines [31].

CR-1 is a multifunctional gene that plays an essential role in embryogenesis as well as cancer growth and progression [11–16]. CR-1 is highly expressed in various human cancers [13]. The truncated form of CR-1 mRNA lacks exons 1 and 2, but the putative open reading frame still contains EGF-like module, cysteine-rich domain and the carboxy-terminal linkage sequence [30, 31]. The truncated form of CR-1 is implicated in human colon carcinomas and hepatic metastases of colon carcinoma, but its function in HCC has not yet been elucidated. Since the short form of CR-1 is the major transcript in HCC and colon cancer cells [30, 31], we hypothesize that it may be involved in cancer progression. However, this hypothesis needs to be investigated in greater detail.

Embryonic genes such as Oct-4 [17–19] and CR-1 [15, 20, 21] are either undetected or expressed at very low levels in normal adult tissues [11–16]. However, CR-1 is overexpressed in several solid tumors [11–16]. Aberrant activation of embryonic genes such as Oct-4 [17–19] and CR-1 [15, 20, 21] is associated with oncogenesis. For example, overexpression of Cripto-1 transgene in the mammary glands of MMTV-CR-1 or WAP-CR-1 transgenic mice promoted mammary epithelial hyperplasia and adenocarcinoma [15, 20] and leiomyosarcoma of the uterus in the MMTV-CR-1 transgenic mice [21]. As reported previously [25–27], our study clearly showed that CR-1 overexpression promoted HCC progression. We demonstrated that hepatocyte-specific overexpression of transgenic CR-1 aberrantly activated several HCC-regulatory signaling pathways. Moreover, we observed deregulated expression of several genes that are key modulators of cellular growth, inflammation, malignant transformation, hepatocellular carcinogenesis, cancer progression, and poor prognosis. However, we did not observe any histological signs of hepatocyte dysplasia or HCC formation in the liver tissues from 3-, 6- or 8-month-old RCLG/Alb-Cre mice. This indicated that constitutive expression of CR-1 transgene alone was not sufficient to promote hepatocarcinogenesis. Our study demonstrated that CR-1 overexpression in the mouse liver and HCC cells significantly activated AKT, Stat3, ERK and JNK pathways, which are closely associated with hepatocyte proliferation, liver regeneration, and hepatocellular carcinogenesis. This suggested that CR-1 overexpression in the mouse liver partly contributed to hepatocyte proliferation and hepatocellular carcinogenesis by modulating the activation of these critical signaling pathways.

CR-1 regulates signaling pathways such as Wnt/β-catenin, AKT, Stat3, MAPK/ERK, TGF-β and Notch [12–15, 22, 25, 55, 56], all of which are implicated in hepatocarcinogenesis, HCC progression, and poor prognosis [9, 10, 57–59]. High levels of phosphorylated AKT and β-catenin were reported in mammary gland tumor tissues from MMTV-CR-1 or WAP-CR-1 transgenic mice [15, 20] and leiomyosarcoma tissues of the uterus in the MMTV-CR-1 transgenic mice [21]. In the present study, CR-1 overexpression in RCLG/Alb-Cre transgenic mouse liver and HCC cells significantly activated Wnt/β-catenin, AKT, MAPK/ERK, Stat3, TGF-β, JNK and Notch pathways. Therefore, our results suggested that hepatocyte-specific overexpression of CR-1 promoted aberrant activation of several signaling pathways involved in premalignant alterations during hepatocarcinogenesis.

Microarray analysis showed that most of the 113 differentially expressed genes found in the liver tissues of RCLG/Alb-Cre mice were related to cellular proliferation, cell cycle, apoptosis, liver regeneration, DNA damage, stress responses, inflammation response, immune escape, defense response, acute-phase response, cellular malignant transformation, oncogenesis or cancer malignant progression, and poor prognosis. These processes are implicated in various liver pathologies including HCC [60–63].

Several genes that were differentially up-regulated (Orm2, Orm3, S100A8 and S100A9) or down-regulated (i.e., DMBT1, Gsta2 and G0S2) in the liver tissues of RCLG/Alb-Cre transgenic mice have been previously associated with hepatocarcinogenesis [37, 38, 64–66]. Transgenic mice with hepatocyte-specific overexpression of DNA binding protein A (dbpA) spontaneously developed HCC at about 1.5 years, but morphological changes in the liver were not observed at 30–40 weeks [37]. Moreover, 31–32-week-old male dbpA transgenic mouse livers showed upregulation of Orm2 (Orosomucoid 2) and Orm3 (Orosomucoid 3), as well as downregulation of G0S2 (G0/G1 switch gene 2) [37]. This was in accordance with our findings in HCC tissues (Figure 7). Gsta2 (glutathione-S-transferase, alpha type 2) mRNA was significantly downregulated in the liver tissues from fenofibrate-induced hepatocarcinogenesis model rats [66]. Deleted in malignant brain tumors 1 (DMBT1) gene was downregulated in RCLG/Alb-Cre transgenic livers (Figure 6 and Supplementary Table 2) and HCC tissues [67]. Deng et al. demonstrated that DMBT1 knockdown enhanced proliferation and malignant transformation of hepatic progenitor cells (HPCs) [38], thereby suggesting an important role for DMB1 in hepatocarcinogenesis. The damage-associated molecular pattern proteins, S100A8 and S100A9, enhanced growth and invasiveness of various cancer cell lines [68] and are implicated in liver, brain, breast, colon, thymus and thyroid cancers [64]. The upregulation of S100A8 and S100A9 correlates with poor differentiation of human liver cancer cells and tissues [64, 65, 69]. The expression levels of S100A8 and S100A9 were elevated in serum and tumor tissue samples from HCC patients [65, 69]. Moreover, higher S100A9 expression was reported in blood serum and tissue samples from HBV-positive patients with HCC compared to those from HBV-negative patients with HCC [69]. Overexpression of S100A8/A9 increased *in vitro* HCC cell survival, growth and invasiveness as well as xenograft tumor size in the mouse model [64, 69]. In the diethylnitrosamine-induced HCC model, S100A8/A9 ablation impaired liver cancer progression by decreasing cancer cell proliferation [64]. S100A8 and S100A9 are NF-μB target genes that synergistically enhance reactive oxygen species (ROS) and survival of HCC cells [64]. Previous studies show that S100A8 and S100A9 proteins form a heterodimer called calprotectin that promoted HCC development and progression [64, 65]. We postulate that increased expression of S100A9 and S100A8 in the liver tissues of RCLG/Alb-Cre transgenic mice promoted hepatocyte transformation and survival against injury.

Pyruvate dehydrogenase complex (PDC) is a key modulator of tricarboxylic acid (TCA) cycle flux in the mitochondria by catalyzing the oxidation of pyruvate into acetyl CoA and NADH, which is required for the TCA cycle and mitochondrial respiration; phosphorylation of PDC is catalyzed in humans by four isozymes of pyruvate dehydrogenase kinase (PDK1, PDK2, PDK3 and PDK4) with about 70% homology [70–75]. PDKs 1–3 are closely associated with metabolism of cancer cells because they inactivate PDC via phosphorylation [73]. PDK4 acts as a potential tumor suppressor because its expression is significantly reduced in lung, ovarian, colon and breast cancers [76–78]. In this study, we demonstrated significantly decreased expression of PDK4 in the RCLG/Alb-Cre transgenic livers (Figure 6 and Supplementary Table 2) and human HCC tissue samples (Figure 7). Moreover, PDK4 silencing significantly increased proliferation, tumorigenicity and invasion of HCC cells (unpublished data). Furthermore, PDK4^-/-^ livers showed enhanced hepatocyte growth, but these effects were inhibited by arsenic treatment [45]. Overall, our data suggested that PDK4 was a potential suppressor of hepatocarcinogenesis in mice and humans.

Several studies have shown that oxidative stress promotes hepatocarcinogenesis [79–81]. In this study, two genes encoding antioxidant proteins, glutathione S-transferase, alpha 1 (Gsta1) and glutathione S-transferase, alpha 2 (Gsta2), were down-regulated and a pro-oxidant protein, vanin 3 (Vnn3), was up-regulated in the RCLG/Alb-Cre transgenic livers (Figure 6 and Supplementary Table 2). Vanin/pantetheinase is highly expressed in several organs such as intestine, liver and kidney; moreover, vanin 1 (Vnn1), vanin 2 (Vnn2) and vanin 3 (Vnn3) induce oxidative stress [82–85]. Down-regulation of oxidant defense genes such as Gsta1 and Gsta2 in the liver was associated with fenofibrate-induced hepatocarcinogenesis in rats [66]. Therefore, we postulate that CR-1 overexpression in the hepatocytes of RCLG/Alb-Cre mice may induce oxidative stress by increasing the expression of Vnn3 and reducing the expression of antioxidant genes, Gsta1 and Gsta2, in RCLG/Alb-Cre liver tissues, thereby enhancing their survival and favoring the onset of hepatocarcinogenesis.

DNA damage plays an integral role in hepatocarcinogenesis [86, 87]. In this study, we observed significant down-regulation in the expression levels of DNA damage response factors such as polo-like kinase 2 (Plk2) and polo-like kinase 3 (Plk3) in the RCLG/Alb-Cre transgenic mouse livers (Figure 6 and Supplementary Table 2) and human HCC tissue samples (Figure 7). Plk2 and Plk3 act as tumor suppressors through their functions in the p53 signaling network and guard cells against various stress signals [88–92]. Plk3 promotes DNA damage-induced cell cycle arrest via the ATM/p53 pathway and Plk3-deficient mice develop tumors [88–92]. Plk2 and Plk3 are involved in checkpoint-mediated cell cycle arrest to protect against accumulation of genetic defects [88–92]. Therefore, the results of our study imply that decreased expression of Plk2 and Plk3 in RCLG/Alb-Cre transgenic liver tissues may promote liver carcinogenesis through increased oxidative stress-related damage and accumulation of genetic mutations in the hepatocytes and their subsequent proliferation.

Chronic liver inflammation promotes HCC because of increased oxidative/nitrosative stress and lipid peroxidation, which oxidatively mutates the genomic DNA [80, 93–96]. In this study, we documented up-regulation of several genes involved in liver inflammation (CD5L, S100A8, S100A9, Timd4 and Rgs16), immune escape (Orm2, Orm3, IGHG1, IGHG2B, IGHG2C and IGKV16-104), acute-phase response (Orm2, Orm3, Saa1, Saa3, Itih3 and Itih4), and immune defense response (Ly6e) (Figure 6 and Supplementary Table 2) in the RCLG/Alb-Cre transgenic livers.

Guerra et al. showed that Api6/AIM/Spa/CD5L was upregulated in HCC, and promoted HCC cell proliferation and survival by binding to HSPA5 (GRP78) [50]. Barcena et al. demonstrated that CD5L regulated liver damage, fibrosis and immune cell content [49]. CD5L overexpression in the alveolar type II epithelial cells (AT II cells) of transgenic mice induced malignant transformation and spontaneous lung adenocarcinoma by inhibiting apoptosis of lung epithelial cells and promoting immune escape [97]. Moreover, CD5L overexpression in AT II cells increased the levels of pro-inflammatory cytokines/chemokines in the bronchoalveolar lavage fluid and serum, promoted expansion of myeloid-derived suppressor cells (MDSC) in lungs and blood; lung MDSCs suppressed *in vitro* T-cell proliferation and activity and reduced *in vivo* levels of T cells *in vivo* following doxycycline-induced CD5L transgene activation [97]. IGHG1 promotes pancreatic cancer cell proliferation and immune evasion [98].

Tumor immune-evasion refers to the ability of cancer cells to circumvent host immune systems and utilize inflammatory factors for tumor growth and progression [99]. T-cell immunoglobulin mucin (TIM) gene family members maintain immune homeostasis by regulating multiple phases of the immune response [99]. Cancer cells evade immunosurveillance via TIM gene family members, which also inhibit inflammation-related tumor progression [99]. Timd4 (T-cell immunoglobulin domain and mucin domain 4; also known as TIM4) plays a critical role in regulating tumor immunosurveillance and anti-tumor immunity [99]. Timd4 overexpression is also associated with increased lung cancer cell proliferation [100].

Acute-phase response (APR) proteins such as Orm2 and Orm3 play an important role in anti-inflammatory and immunomodulatory responses that are initiated against infections, physical trauma, or malignancies [36]. Orm2 is frequently down-regulated in HCC tissues and negatively correlates with tumor progression and intrahepatic metastasis [36]. The serum levels of another APR protein, ITIH4, are elevated in HCC patients during acute phase; moreover, ITIH4 is up-regulated by interleukin-6 in HepG2 cells [101]. Collectively, our findings suggest that CR-1 regulates several inflammatory and host immunity factors that are involved in HCC growth and progression.

In conclusion, our study demonstrated that SF-CR-1 mRNA was predominantly expressed in most HCC tissues and cell lines. However, the biological function of SF-CR-1 mRNA in HCC progression requires further investigation. We also demonstrated that hepatocyte-specific overexpression of CR-1 in transgenic mice deregulated several signaling pathways and genes involved in hepatocarcinogenesis. However, hepatocyte-specific CR-1 overexpression alone was not sufficient to initiate hepatocarcinogenesis in mice. Therefore, the exact role of CR-1 in HCC remains to be further explored, plausibly using the carcinogen (DEN: diethylnitrosamine)-induced HCC model.

## MATERIALS AND METHODS

### Cell lines and cell culture

HEK293T cells were purchased from the American Type Culture Collection (ATCC). The human HCC cell lines (HepG2 and BEL-7402) were obtained from the Cell Bank of the Chinese Academy of Sciences (Shanghai, China). These cell lines were cultured in Dulbecco’s modified Eagle’s medium (DMEM) (high glucose) supplemented with 10% fetal bovine serum (FBS) in a humidified incubator maintained at 5% CO_2_ and 37°C.

### Clinical specimens

Fresh primary HCC specimens and tumor-adjacent noncancerous tissues were collected from HCC patients that underwent surgery at the Sun Yat-sen Memorial Hospital, Sun Yat-sen University (Guangzhou, China). We obtained informed consent from all patients. The inclusion criteria were: (1) a definite pathological diagnosis of HCC and (2) no other anti-cancer treatment before surgery. We extracted total RNA and protein from the fresh-frozen specimens for qRT-PCR and western blot, respectively. We then prepared formalin-fixed paraffin-embedded blocks from the fresh-frozen HCC and adjacent non-tumor liver specimens. This study was approved by the Medical Ethics Committee of Southern Medical University.

### RT-PCR and quantitative real-time PCR (qRT-PCR)

We isolated total RNA and performed reverse transcription, RT-PCR and qRT-PCR using protocols as previously described [3, 16, 102–105]. For RT-PCR, we amplified two distinct regions of the CR-1 mRNA based on the previously published CR-1 cDNA sequence [30, 31] using CR-1-specific primers. The CR-1 specific primers were: UN-A forward primer (nucleotides 375–398): 5′-ACCTGGCCTTCAGAGATGACAGCA-3′, UN-B reverse primer (nucleotides 656–680): 5′-ATGCCTGAGGAAAGCAGCGGAGCT-3′ and UN-D forward primer (nucleotides 248–266): 5′-AAAGCTATGGACTGCAGGA-3′ (Figure 1A). UN-A/UN-B and UN-D/UN-B primer sets yielded 305 bp and 432 bp sized PCR products, respectively (Figure 1A). GAPDH was used as internal control. The primer pairs for amplification of GAPDH are listed in Supplementary Table 3. The primer pairs used in qRT-PCR are listed in Supplementary Tables 4 and 5. GAPDH was used as internal control. All samples were normalized to internal controls. The relative fold changes in specific mRNA levels were calculated using the 2^-ΔΔCt^ method.

### Western blotting

Western blot analysis was performed as previously described [3, 16, 102–105]. GAPDH or β-actin were used as loading controls. The primary antibodies used in this study are listed in Supplementary Table 6.

### Histological analysis and immunohistochemistry (IHC)

Histological analysis and immunohistochemical staining were performed as previously described [3, 16, 106–109]. The antibodies used in the study and the experimental conditions are summarized in the Supplementary Table 6.

### Lentivirus production and transduction

The human CR-1 lentiviral shRNA vector (pLV-shCR-1), empty lentiviral vector expressing scrambled shRNA (pLV-shSCR), lentiviral CR-1 expression vector (pLV-CR-1), and empty lentiviral vector (pLV-con) were generously provided by Prof. Peter C. Gray (The Salk Institute for Biological Studies, USA) [110]. The lentiviral packaging plasmids, psPAX2 and pMD2.G, were kindly provided by Dr. Didier Trono (University of Geneva, Geneva, Switzerland). We infected BEL-7402 and HepG2 cells with pLV-con, pLV-CR-1, pLV-shSCR, and pLV-shCR-1, respectively, in combination with lentiviral packaging plasmids (psPAX2 and pMD2.G) to generate stable cell lines, and then isolated recombinant lentiviruses, namely LV-con, LV-CR-1, LV-shSCR and LV-shCR-1 as previously described [105, 111].

### Colony formation assay

Colony formation assay was performed as previously described [3, 104].

### Transwell migration and Boyden invasion assays

Transwell migration and Boyden invasion assays were performed as described previously [3, 104, 109].

### Tumor xenografts in nude mice

Three- to four-week old female BALB/c nude mice were obtained from the Medical Laboratory Animal Center of Guangdong Province. We subcutaneously injected CR-1-overexpressing or vector-expressing BEL-7402 cells (1 × 10^6^ cells) into the right or left dorsal thigh of mice (*n* = 7), respectively. The tumor size parameters were measured every 2 days using a caliper slide rule and tumor volumes were calculated using the following formula: Tumor volume = (D × d^2^)/2, where ‘D’ is the longest diameter and ‘d’ is the shortest diameter. The mice were sacrificed after 20 days. The subcutaneous tumors were harvested, weighed, fixed overnight in 4% paraformaldehyde, dehydrated, paraffin-embedded, and sectioned. The animal experiments were carried out strictly according to the recommendations in the Guide for the Care and Use of Laboratory Animals of the Southern Medical University. The protocols for animal experiments were approved by the Committee on Ethics of Animal Experiments of the Southern Medical University. All surgeries were performed under sodium pentobarbital anesthesia. All efforts were made to minimize the suffering of animals.

### Generation of RCLG transgenic mice

The pCI-CR-1 vector [112] was generously provided by Dr. David S. Salomon (Center for Cancer Research, National Cancer Institute, USA). The pCAG-RLG vector [113, 114] was generously provided by Prof. Manuela Martins-Green (University of California, USA). We PCR amplified a 600 bp fragment of CR-1 cDNA from the pCI-CR-1 as previously described [112] and cloned it into the *Sma* I site of the pCAG-RLG plasmid [113, 114]. The RCLG transgenic construct was confirmed by PCR, DNA sequencing and enzyme digestion analysis (data not shown) and designated as pCAG-RCLG (Supplementary Figure 1A).

RCLG transgenic mice were generated by microinjecting DNA into the pronuclei of fertilized embryos as previously described [115]. Three days after birth, the off-springs were screened to identify potential RCLG transgenic founders using mRFP assay with the IVIS Lumina II imaging system (Xenogen Corp., Alameda, CA, USA) and subsequently confirmed by PCR-based genotyping.

### Whole-animal (*in vivo*) and organ (*ex vivo*) fluorescence imaging

Fluorescence imaging of mRFP (monomeric red fluorescent protein) in the whole-animal and organs was performed using stereo fluorescence microscope (Nikon, AZ100) or the Xenogen IVIS Lumina II Imaging System according to the protocols described previously [16, 102, 104, 116–118].

### *In vivo* and *ex vivo* optical imaging of firefly luciferase (Luc) activity

We purchased homozygous Alb-Cre mice (B6. Cg-Tg (Alb-cre) 21Mgn/J) from the Model Animal Research Center of Nanjing University and crossed them with the RCLG mice to obtain RCLG/Alb-Cre double transgenic mice. Firefly luciferase (Luc) expression was observed specifically in the liver of the RCLG/Alb-Cre double transgenic mice using the non-invasive *in vivo* bioluminescence imaging and quantified using the IVIS Lumina II imaging system. The protocols for bioluminescence imaging to detect Luc activity in the whole-animal (*in vivo*) and the dissected organs (*ex vivo*) using the IVIS system were as previously described [3, 16, 102, 104, 116–118].

### PCR genotyping

We performed PCR analysis using the tail genomic DNA from the mice to further confirm the RCLG/Alb-Cre double transgenic mice. The specific forward primer (FP) and reverse primer (RP) sequences for the Luc and Cre genes are shown in Supplementary Table 7.

### Microarray analysis

We used 32K mouse genome microarray (Beijing Capital biology company, China) to perform mRNA expression microarray analysis as previously described [28]. Total RNA was extracted from the livers of control and RCLG/Alb-Cre transgenic mice. The sequence hybridization and data analysis were performed by Capital Bio Corp. (Bejing, China).

### Statistical analysis

The data are presented as means ± SEM. Statistical analysis was performed using the SPSS 13.0 software package and Graphpad 5.0 software. Two-tailed Student’s *t* test was used to compare data between two independent groups. Statistical significance was set at ^*^P < 0.05, ^**^P < 0.01 and ^#^*P* < 0.001.

## Supplementary Materials

Supplementary Figures

Supplementary Tables
