# Supplementary material for: Liver-specific over-expression of Cripto-1 in transgenic mice promotes hepatocyte proliferation and deregulated expression of hepatocarcinogenesis-related genes and signaling pathways
Source: Aging (Albany NY). 2021 Sep 13;13(17):21155–90. doi: 10.18632/aging.203402 (PMC8457585; doi:10.18632/aging.203402)
Supplement: Supplementary Figures [file aging-13-203402-s001.pdf]

SUPPLEMENTARY FIGURES

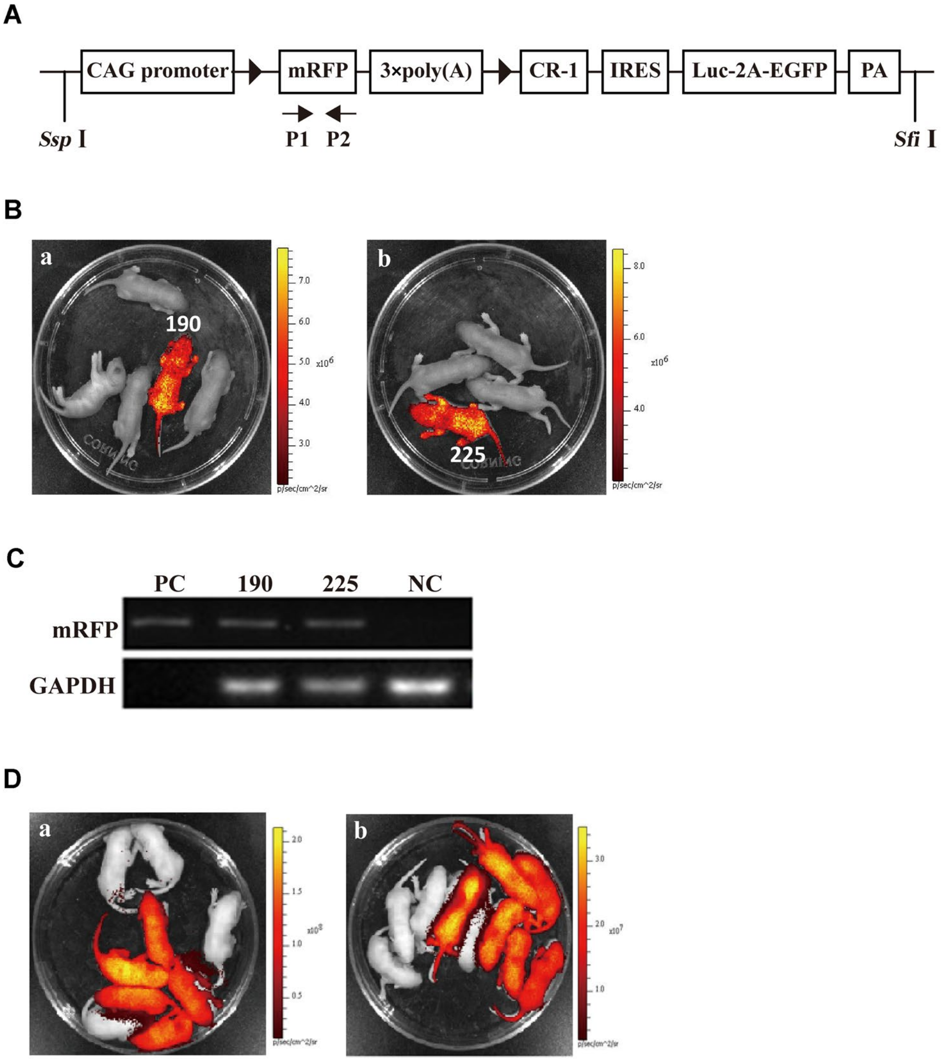

Supplementary Figure 1. Generation of RCLG transgenic mice. (**A**) Schematic diagram of the RCLG transgenic construct used to generate RCLG transgenic mice. A potent, ubiquitous CMV/β-actin promoter in the vector pRCLG was used to drive a series of cassettes, including a floxed mRFP followed by a triple transcription-stopping polyA sequence (3×PolyA) and a downstream internal ribosome entry site (IRES)-based bicistronic transcript, including open-reading frames of human Cripto-1 and a multifunctional marker consisting of firefly Luc fused to eGFP with a transmembrane-localizing domain (Luc-TMeGFP). The primer pair P1/P2 represented by small arrows were used in PCR analysis of genotype to detect reporter transgene mRFP. Only mRFP will be transcribed and expressed properly from this construct, while Cre-mediated recombination occurs, the floxed mRFP+3×PolyA is excised, and the downstream, bicistronic transcript is activated. The multifunctional marker will be expressed, replacing mRFP in Cre-activated cells. The construct map is not drawn to the scale. Abbreviations: CAG promoter: CMV early enhancer/chicken β actin promoter; mRFP: monomeric red fluorescent protein; Luc: firefly luciferase; EGFP: enhanced green fluorescent protein; pA: polyadenylation signal; ▶: *lox* P site. (**B**) Screening RCLG transgenic founders by *in vivo* non-invasive fluorescence imaging. Two foster mothers gave birth to five and four F0 pups, respectively; two mRFP-positive RCLG transgenic mice (referred to as 190# and 225#) with strong red fluorescence were found via mRFP assay by using the Xenogen IVIS Lumina Imaging System 2–3 days after birth (Supplementary Figure 1B-a, b). (**C**) mRFP-positive founders verified for RCLG transgene presence by PCR analysis. Two mRFP-positive mice (i.e., 190# and 225#) and one mRFP-negative mice (i.e., 1109#) were individually analyzed by PCR for the genomic integration of transgene with tail biopsy-derived DNA from mice (190# and 225#). PCR products were amplified by the primer pair P1/P2 (specific for mRFP) shown in Supplementary Figure 1A. The sequences of the forward primer (P1) and reverse primer (P2) used to amplify a 339-bp fragment of the RCLG transgene were: 5'-GGGAGCGCGTGATGAAC-3' (P1) and 5'-CGTTGTGGGAGGTGATGTC-3' (P2). lane PC: positive control (pRCLG as template); lane NC: negative control using genomic DNA from WT mouse as template. Data are representative of three independent PCR experiments that yield similar results. (**D**) F1 progeny inherit and express mRFP transgene from three founders Offspring shown in Supplementary Figure 1D-a, b were derived from the mating between founder 190# or 225# and wildtype mouse, respectively.

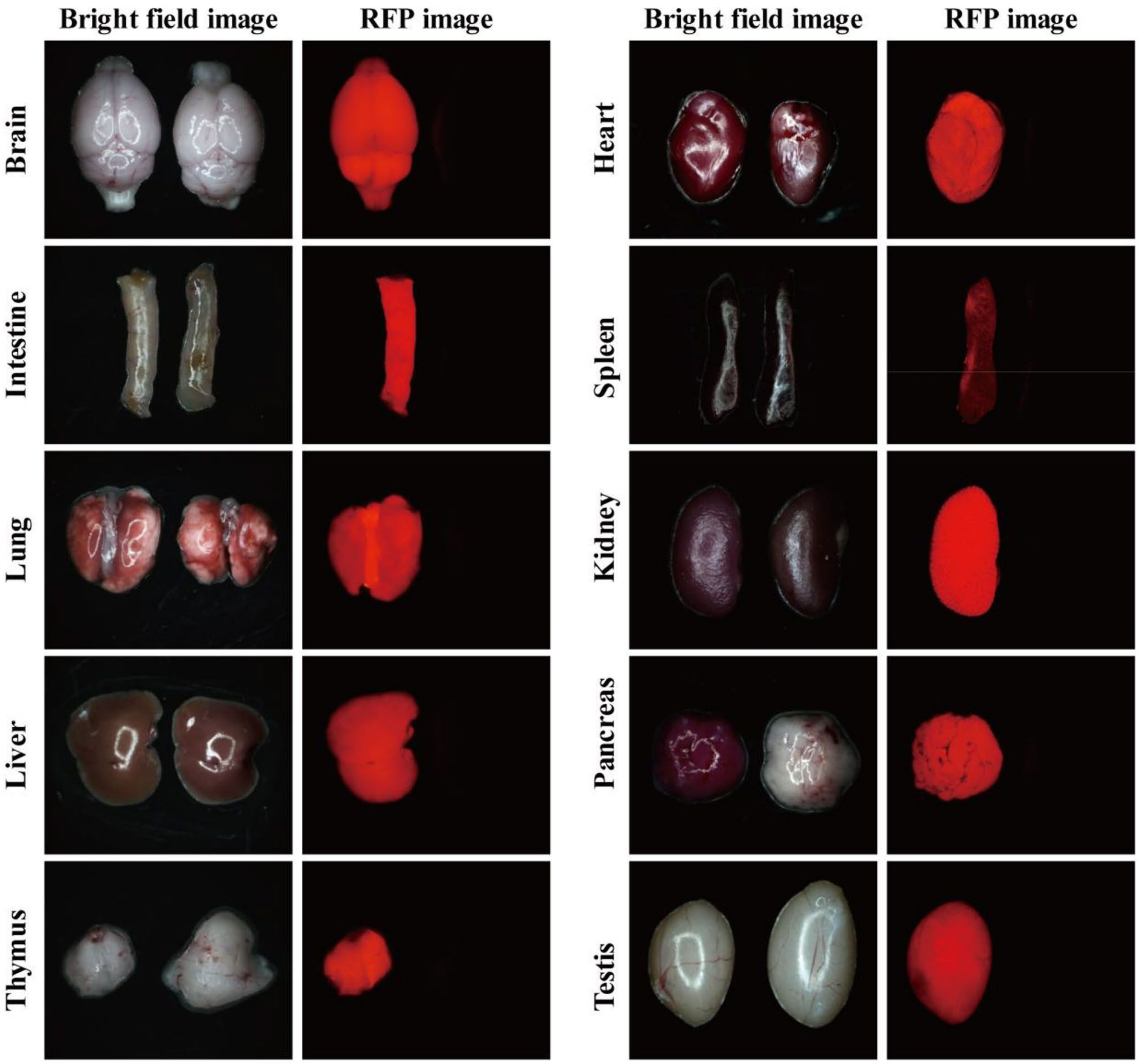

Supplementary Figure 2. mRFP expression in postnatal organs of RCLG transgenic mice. mRFP expression in the postnatal organs of RCLG transgenic mice was detected under stereo fluorescence microscope (Nikon, AZ100). The right organ samples in each figure were obtained from one non-transgenic littermate, while the left organ samples in each figure were obtained from one RCLG transgenic mouse. Pancreas from RCLG transgenic mice (the right samples in each figure) can be distinguished from their wildtype littermates according to their deep red color under daylight.

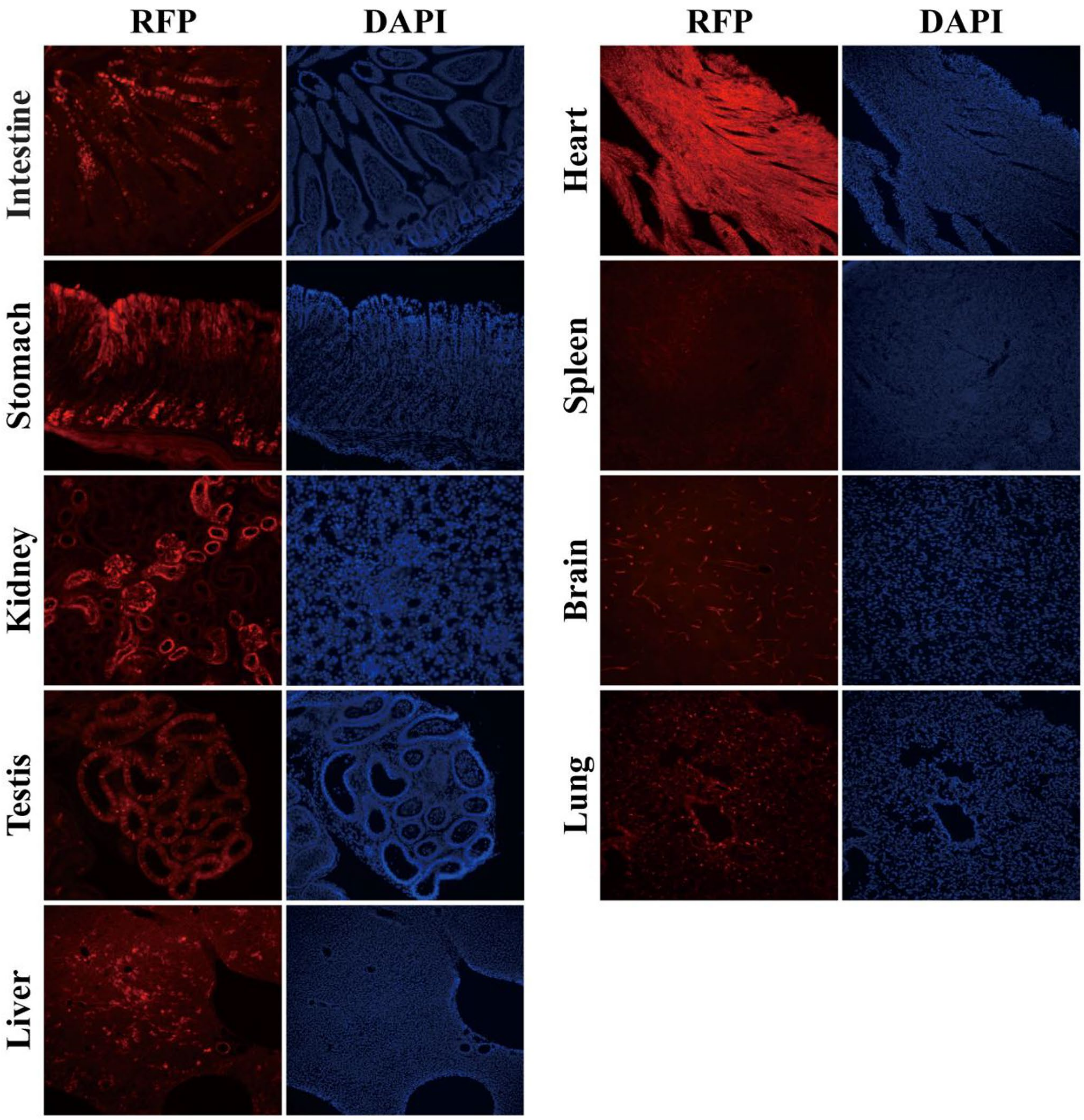

Supplementary Figure 3. Histological analysis of mRFP expression in different tissues of solid organs of RCLG transgenic mice. Frozen tissue sections from RCLG transgenic mice were directly visualized for red fluorescence under upright fluorescence microscope (Nikon, Eclipse 80i), while the nuclei in all panels were shown with DAPI staining (Blue channel).

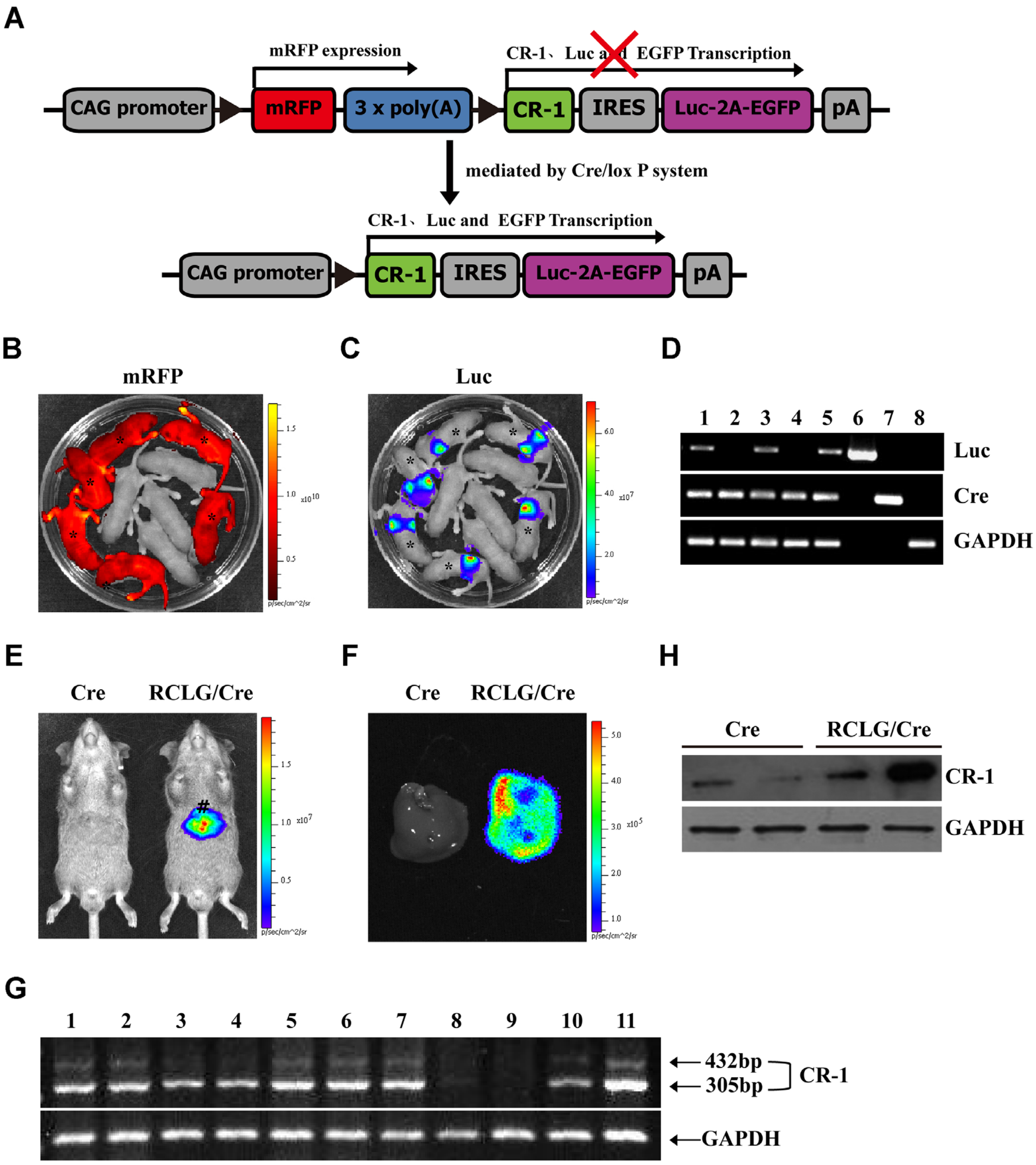

Supplementary Figure 4. Liver-specific overexpression of CR-1 in transgenic mice mediated by Cre/*lox* P system. (**A**) Strategy for liver-specific expression of CR-1 transgene using Cre/*lox* P system. In the absence of Cre-mediated recombination, only mRFP will be transcribed, while CR-1 and Luc (Luciferase) transgene expression is prevented by STOP sequence flanked by *lox* P sites. When Cre-mediated recombination occurs in mouse liver, the floxed mrfp+3×PolyA is excised, and CR-1 and Luc transgene expression is activated in a liver-restricted pattern in RCLG/Alb-Cre double transgenic mice. Other details as in Supplementary Figure 1A. (**B**–**C**) Whole-body fluorescence (**B**) and bioluminescence (**C**) imaging for newborn offspring derived from mating heterozygous RCLG transgenic mice with homozygous Alb-Cre mice. (**D**) mRFP-positive (in Supplementary Figure 4B) or Luc-positive (in Supplementary Figure 4C) newborn offspring verified for Luc and Cre transgene presence by PCR-based genotyping analysis. (**E**) *In vivo* luciferase imaging in the liver of both adult RCLG/Alb-Cre mouse and the control littermate developing from these offspring shown in Supplementary Figure 4B, 4C. (**F**) *Ex vivo* imaging of Luc expression in liver obtained from same mouse shown in Figure 3E. (**G**) RT-PCR for human CR-1 transgene isoform expression in liver from double transgenic mouse (RCLG/Alb-Cre^{Tg}) and littermate control. The primer couples UN-D/UN-B and UN-A/UN-B yield PCR products of 432 bp and 305 bp (Figure 1), respectively. Lane 1: 3-week-old Luc-positive mouse; Lane 2 and 3: 3-month-old Luc-positive mouse; Lane 4 and 5: 6-month-old Luc-positive mouse; Lane 6 and 7: 8-month-old Luc-positive mouse; Lane 8: Luc-negative mouse; Lane 9: wild-type mouse; Lane 10: adjacent non-tumorous human liver tissues (N); Lane 11: human HCC tissue (T). Other details as in Figure 1A. (**H**) The expression of Cirpto-1 protein in livers from double transgenic mouse (RCLG/Alb-CreTg) and littermate control examined by Western blotting.

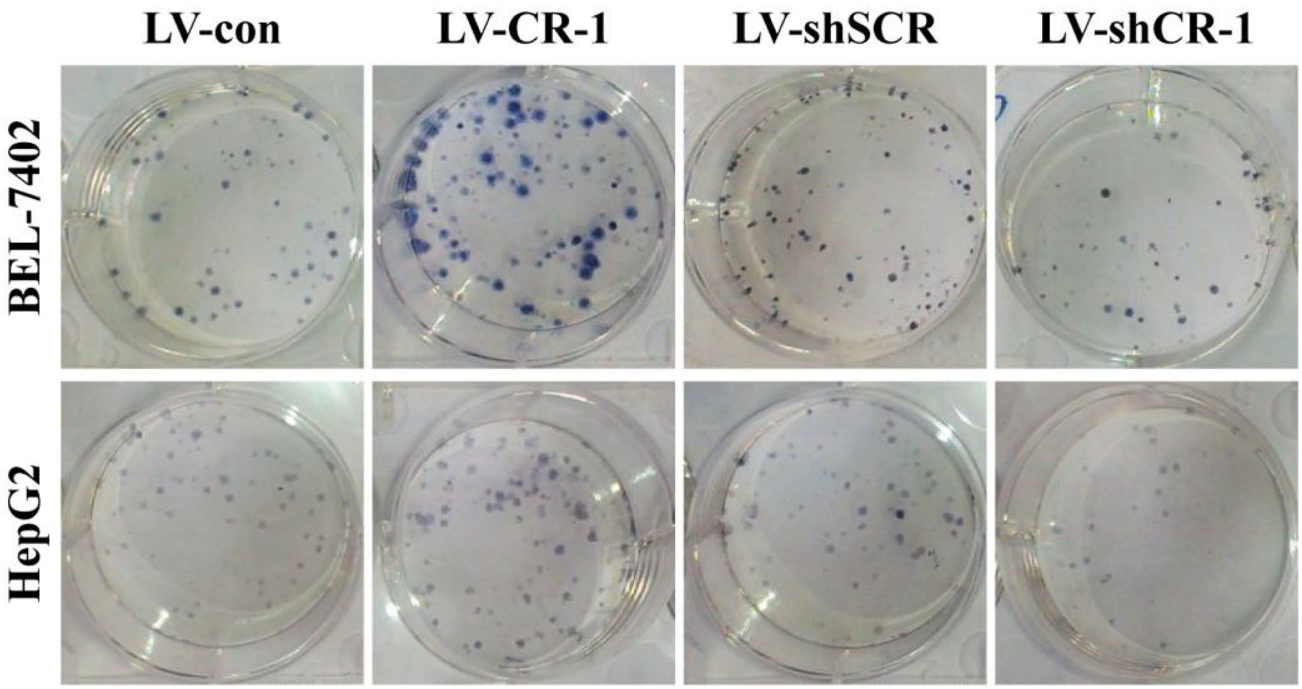

Supplementary Figure 5. Representative photographs of the proliferation activities of CR-1- or shCR-1-expressing Bel-7402 and HepG2 cells based on colony formation assay. The statistical data on the proliferation activities of the indicated cancer cells were provided in Figure 4C.

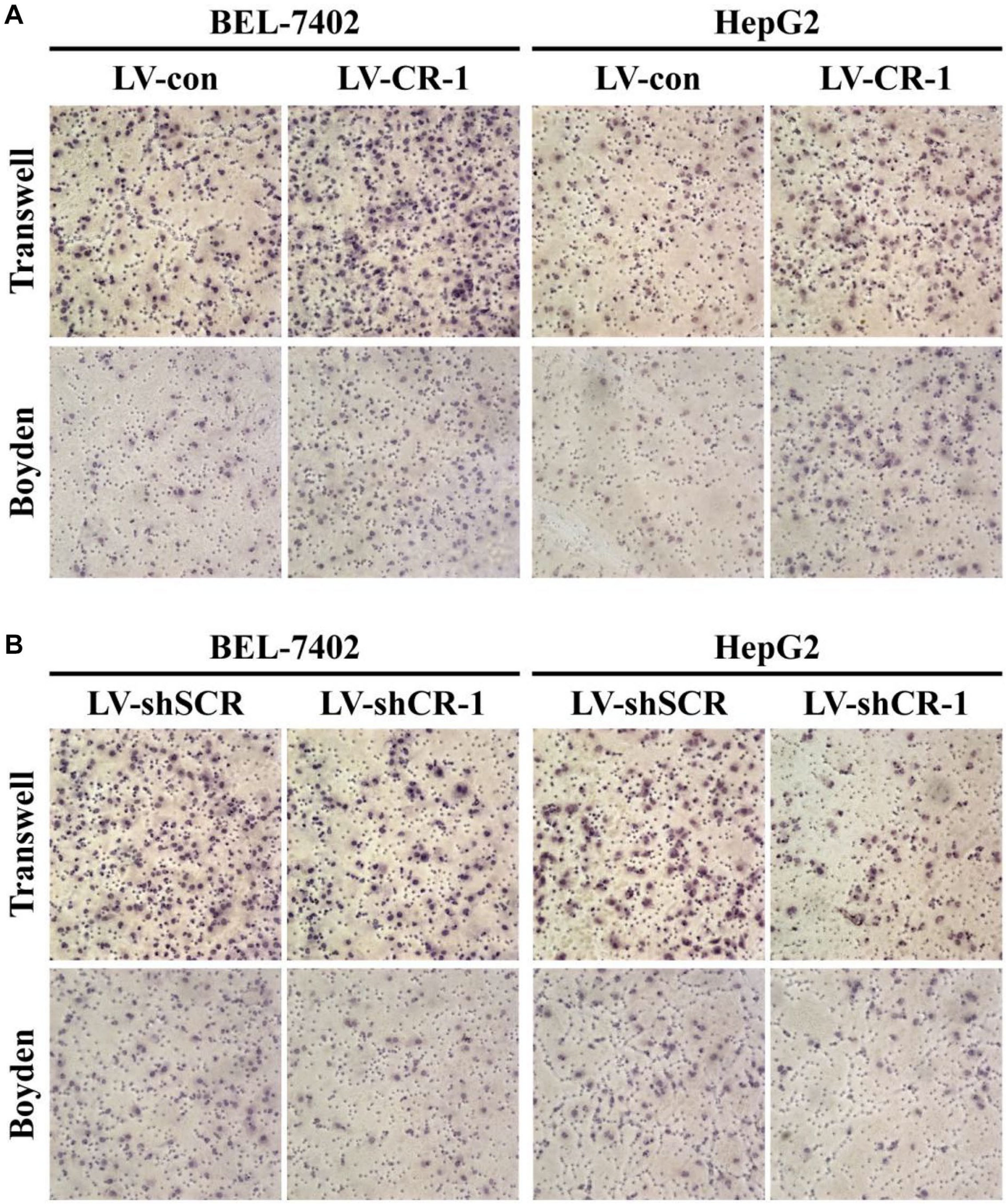

Supplementary Figure 6. Representative photographs of the motility and invasion ability of CR-1-or shCR-1-expressing Bel-7402 and HepG2 cells based on transwell migration and Boyden invasion assays, respectively. (**A**) The motility and invasion ability of CR-1-expressing Bel-7402 and HepG2 cells based on transwell migration and Boyden invasion assays. (**B**) The motility and invasion ability of shCR-1-expressing Bel-7402 and HepG2 cells based on transwell migration and Boyden invasion assays. The statistical data on the motile and invasive activities of the indicated cancer cells were provided in Figure 4D.

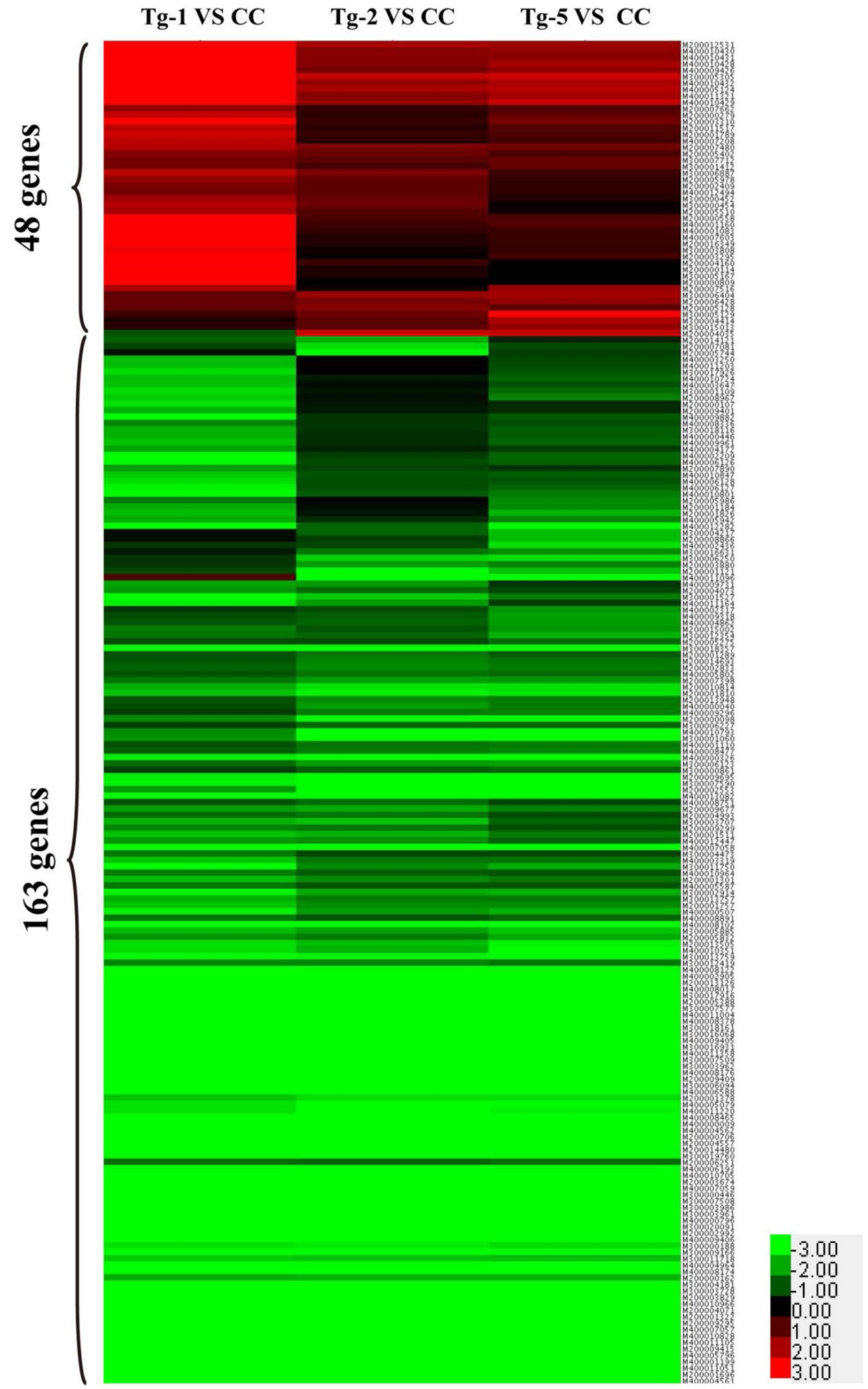

Supplementary Figure 7. Class comparison and hierarchical clustering analysis of differentially expressed genes between RCLG/Alb-Cre and control mouse liver. Tg-1, Tg-2 and Tg-5 represented the total RNA (used in microarray experiment) isolated from the livers of three 4-month-old RCLG/Alb-Cre transgenic mice, while cc represented the pooled total RNA (used in microarray experiment) isolated from the livers of three control littermates. Equal amounts of total RNA from each control liver vole were pooled to prepare cc. Tg-1 vs cc: Tg-1 compared to pooled cc; Tg-2 vs cc: Tg-2 compared to pooled cc; Tg-5 vs cc: Tg-5 compared to pooled cc. Only genes showing a fold change of more than 2 and a *t* test *P* value of less than 0.05 were included in the analysis. Red indicates increased expression; blue indicates reduced expression The mRNA microarray analysis showed that 211 mRNAs were differentially expressed between RCLG/Alb-Cre and control mouse liver.
