# Supplementary material for: Liver-specific over-expression of Cripto-1 in transgenic mice promotes hepatocyte proliferation and deregulated expression of hepatocarcinogenesis-related genes and signaling pathways
Source: Aging (Albany NY). 2021 Sep 13;13(17):21155–90. doi: 10.18632/aging.203402 (PMC8457585; doi:10.18632/aging.203402)
Supplement: Supplementary Tables [file aging-13-203402-s002.pdf]

SUPPLEMENTARY TABLES

Supplementary Table 1. Differentially expressed genes in liver between control and RCLG/Alb-Cre transgenic mice.

Differential expression	Number of genes	Fold difference (RCLG/Alb-Cre vs con)
Up-regulated	48	2.0181–7.8
Down-regulated	163	0.0045–0.499
Total	211	

Supplementary Table 2. HCC-related genes differentially expressed between control and RCLG/Alb-Cre transgenic mice (average of three biological replicates >2 fold-change, *t*-test $p < 0.05$).

Gene symbol	Description (Full name)	Fold difference (RCLG/Alb-Cre vs con)
Lcn2	lipocalin 2	7.8899
IGKV16-104	Immunoglobulin Kappa light chain V gene segment	6.5300
IGHG1	Immunoglobulin heavy chain C gene segment	6.4945
Orm2	orosomucoid 2	5.3184
IGHG2B	Immunoglobulin heavy chain C gene segment	3.5573
Slpi	secretory leukocyte peptidase inhibitor	3.5081
Rgs16	regulator of G-protein signaling 16	3.1999
Dffa	DNA fragmentation factor, alpha subunit	3.0666
Saa1	serum amyloid A 1	2.9429
Tmem176b	transmembrane protein 176B	2.8218
Orm3	orosomucoid 3	2.7805
IGHG2C	Immunoglobulin heavy chain C gene segment	2.7015
S100a8	S100 calcium binding protein A8 (calgranulin A)	2.6769
Ocel1	occludin/ELL domain containing 1	2.6553
Ly6e	lymphocyte antigen 6 complex, locus E	2.5509
Itih3	inter-alpha trypsin inhibitor, heavy chain 3	2.5345
Nr0b2	nuclear receptor subfamily 0, group B, member 2	2.5118
Saa3	serum amyloid A 3	2.4707
Spp1	secreted phosphoprotein 1	2.4077
Gats	opposite strand transcription unit to Stag3	2.3934
Tmem176a	transmembrane protein 176A	2.3734
Tsc22d3	TSC22 domain family 3	2.3724
Cp	ceruloplasmin	2.2608
C4b	complement component 4B (Childo blood group)	2.2089
Gm1381	gene model 1381, (NCBI)	2.1777
Itih4	inter alpha-trypsin inhibitor, heavy chain 4	2.1524
S100a9	S100 calcium binding protein A9 (calgranulin B)	2.124
Vnn3	vanin 3	2.0685
Cd5l	CD5 antigen-like	2.0606
Timd4	T-cell immunoglobulin and mucin domain containing 4	2.0181
Plk2	polo-like kinase 2 (Drosophila)	0.6407
Casp4	caspase 4, apoptosis-related cysteine peptidase	0.6273
Casp8	caspase 8	0.5655
Ablim1	actin-binding LIM protein 1	0.4990
Lgals4	lectin, galactose binding, soluble 4	0.4957
Tmed5	transmembrane emp24 protein transport domain containing 5	0.4910
Plk3	polo-like kinase 3 (Drosophila)	0.4899
Sgk2	serum/glucocorticoid regulated kinase 2	0.4886
Tmem140	transmembrane protein 140	0.4599
Inmt	indolethylamine N-methyltransferase	0.4491
Ifi47	interferon gamma inducible protein 47	0.4418
Grpel2	GrpE-like 2, mitochondrial	0.4409

Hspb1	heat shock protein 1	0.4297
Akr1c19	aldo-keto reductase family 1, member C19	0.4215
Alas2	aminolevulinic acid synthase 2, erythroid	0.4088
Hddc3	HD domain containing 3	0.4063
Serpine2	serine (or cysteine) peptidase inhibitor, clade E, member 2	0.4060
Hip1	huntingtin interacting protein 1	0.3987
Sucnr1	succinate receptor 1	0.3876
Acaa1b	acetyl-Coenzyme A acyltransferase 1B	0.3842
Slc22a5	solute carrier family 22 (organic cation transporter), member 5	0.3819
Chrna2	cholinergic receptor, nicotinic, alpha polypeptide 2 (neuronal)	0.3782
Gdf15	growth differentiation factor 15	0.3750
Mod1	malic enzyme, supernatant	0.3574
Slc39a5	solute carrier family 39 (metal ion transporter), member 5	0.3535
Cish	cytokine inducible SH2-containing protein	0.3429
Efnb1	ephrin B1	0.2956
Amigo1	adhesion molecule with Ig like domain 1	0.2862
Xlr3a	X-linked lymphocyte-regulated 3A	0.2847
Vipr2	vasoactive intestinal peptide receptor 2	0.2772
Serinc2	serine incorporator 2	0.2711
Zfhx1a	zinc finger homeobox 1a	0.2509
Gsta1	glutathione S-transferase, alpha 1 (Ya)	0.2440
Asns	asparagine synthetase	0.2405
Mthfd2	methylenetetrahydrofolate dehydrogenase (NAD+	0.2340
Gsta2	glutathione S-transferase, alpha 2 (Yc2)	0.2332
Amy1	amylase 1, salivary	0.2199
Foxred2	FAD-dependent oxidoreductase domain containing 2	0.1900
Reg3g	regenerating islet-derived 3 gamma	0.1568
G0s2	G0/G1 switch gene 2	0.1553
Ube2c	ubiquitin-conjugating enzyme E2C	0.1525
Klk15	kallikrein related-peptidase 15	0.1324
Pdk4	pyruvate dehydrogenase kinase, isoenzyme 4	0.0917
Crtc1	CREB regulated transcription coactivator 1	0.0863
Smpx	small muscle protein, X-linked	0.0859
Cttn	cortactin	0.0856
Teddm1	transmembrane epididymal protein 1	0.0807
Lmo6	LIM domain only 6	0.0751
Slc38a5	solute carrier family 38, member 5	0.0671
Klk1b27	kallikrein 1-related peptidase b27	0.0609
Reg2	regenerating islet-derived 2	0.0525
Klk1b4	kallikrein 1-related pepidase b4	0.0475
Klk1b5	kallikrein 1-related peptidase b5	0.0418
Klk1	kallikrein 1	0.0400
Spink3	serine peptidase inhibitor, Kazal type 3	0.0380
Prss2	protease, serine, 2	0.0378
Klk1b24	kallikrein 1-related peptidase b24	0.0359
Nupr1	nuclear protein 1	0.0352
Ins2	insulin II	0.0319
Klk1b8	kallikrein 1-related peptidase b8	0.0318
Klk1b26	kallikrein 1-related petidase b26	0.0310
Ihpk3	inositol hexaphosphate kinase 3	0.0297
Aqp12	aquaporin 12	0.0294
Pnlip	pancreatic lipase	0.0274
Tff2	trefoil factor 2 (spasmolytic protein 1)	0.0271
Pla2g1b	phospholipase A2, group IB, pancreas	0.0261
Reg1	regenerating islet-derived 1	0.0260
Ctrc	chymotrypsin C (caldecrin)	0.0256

Klk1b3	kallikrein 1-related peptidase b3	0.0243
Pdia2	protein disulfide isomerase associated 2	0.0242
Pnliprp2	pancreatic lipase-related protein 2	0.0239
Prss3	protease, serine, 3	0.0234
Klk1b11	kallikrein 1-related peptidase b11	0.0182
Serpini2	serine (or cysteine) peptidase inhibitor, clade I, member 2	0.0181
Amy2	amylase 2, pancreatic	0.0181
Rnase1	ribonuclease, RNase A family, 1 (pancreatic)	0.0158
Pnliprp1	pancreatic lipase related protein 1	0.0145
Tmed6	transmembrane emp24 protein transport domain containing 6	0.0136
Gp2	glycoprotein 2 (zymogen granule membrane)	0.0128
Klk1b21	kallikrein 1-related peptidase b21	0.0123
Cuzd1	CUB and zona pellucida-like domains 1	0.0115
Dmbt1	deleted in malignant brain tumors 1	0.0097
Cpa2	carboxypeptidase A2, pancreatic	0.0086

Note: some genes showing a fold change of more than 1.5 and less than 2 and a *t* test *P* value of less than 0.05 were also shown in this table.

Supplementary Table 3. Primers for RT-PCR analysis.

Gene	Forward primer (5′–3′)	Reverse primer (5′–3′)
GAPGH	ACCCAGAAGACTGTGGATGG	TCTAGACGGCAGGTCAGGTC
UN-A	ACCTGGCCTTCAGAGATGACAGCA	
UN-B		ATGCCTGAGGAAAGCAGCGGAGCT
UN-D	AAAGCTATGGACTGCAGGA	

Supplementary Table 4. Primers for qRT-PCR analysis (human).

Gene	Forward primer (5′–3′)	Reverse primer (5′–3′)
GAPGH	ACCCAGAAGACTGTGGATGG	TCTAGACGGCAGGTCAGGTC
Cripto-1	CACGATGTGCGCAAAGAGA	TGTAAATGCTGGCACGGTCA
G0S2	CGCCGTGCCACTAAGGTC	GCACACAGTCTCCATCAGGC
PDK4	AACACCAGGAAAATCAGCC	AAAACCAGCCAAAGGAGC
Plk2	CTACGCCGCAAAAATTATTCCTC	TCTTTGTCCTCGAAGTAGTGGT
Plk3	TTTTCGCACCACTTTGAGGAC	GAGGCCAGAAAGGATCTGCC
Tmem176a	ACAGCCGACAGTGATGAGATG	GGTGTAGTCGCGGATGTAGAAA
Tmem176b	ATGACGCAAAACACGGTGATT	GCAGTTGTGTCAAAGCTGACT
Rnase1	ACTGTAACCAAATGATGAGGCG	GTACCTGGAGCCGTTTGTCA
Klk1	TGTGTGGACCTCAAAATCCTGC	GTAGCCCCATGATGTGACACC

Supplementary Table 5. Primers for qRT-PCR analysis (mouse).

Gene	Forward primer (5′–3′)	Reverse primer (5′–3′)
GAPGH	AGGTCGGTGTGAACGGATTTG	GGGGTCGTTGATGGCAACA
Notch1	CACCCATGACCACTACCCAGTT	CCTCGGACCAATCAGAGATGTT
IL-6	ACCAGAGGAAATTTTCAATAGGC	TGATGCACTTGCAGAAAACA
TGF-β1	GTGGAAATCAACGGGATCAG	ACTTCCAACCCAGGTCCTTC

IL-1	GGTCAAAGGTTTGGAAGCAG	TGTGAAATGCCACCTTTTGA
G0S2	AGTGCTGCCTCTCTTCCCAC	TTTCCATCTGAGCTCTGGGC
PDK4	TTTGGTGGAGTTCCATGAGAA	GAACTTTGACCAGCGTGTCT
Acaa1b	CAGGACGTGAAGCTAAAGCCT	CTCCGAAGTTATCCCCATAGGAA
Rnase1	CTGCAACCAAATGATGAAACGC	CCTTCAGGTGGCAGTCAGTG
CD5L	GATCGTGTTTTTCAGAGTCTCCA	TGCAGTCAACCCCTTGAATAAG
Itih3	AAGGGCAAGTACGAGATGTACC	CCCTGTGGCTCGAAGATGT
Ly6e	TGCGGGCTTTGGGAATGTC	CGGATGCCACACCGAGATT
Foxred2	TGTGGAGGGGTACGAGTCTG	TGTTTTCTGCCGTCTCGAAGG
Klk1	CAATGTGGGGGTATCCTGCTG	GGGTATTCATATTTGACGGGTGT

Supplementary Table 6. List of antibodies and suppliers used for immunoblotting and immunohistochemistry.

Antibody	Isotype	Suppliers	Cat. No
Cripto-1	Rabbit IgG	Abcam	ab60626
PDK4	Rabbit IgG	Proteintech	12949-1-AP
Ki67	Rabbit IgG	Abcam	ab16667
BrdU	Mouse IgG	GE Healthcare	RPN202
P-stat3(P-Y705)	Rabbit IgG	Abcam	ab76315
stat3	Rabbit IgG	Abcam	ab32500
Phospho-p44/42 MAPK (Erk1/2) (Thr202/Tyr204)	Rabbit IgG	Cell Signaling Technology (CST)	9101S
p44/42 MAPK (Erk1/2)	Rabbit IgG	CST	9102S
P-AKT(S473)	Rabbit IgG	CST	4060S
AKT (pan)	Rabbit IgG	CST	4685S
P-GSK-3-beta(S9)	Rabbit IgG	CST	9323S
GSK-3-beta	Rabbit IgG	CST	9315S
Phospho-SAPK/JNK (Thr183/Tyr185)	Rabbit IgG	CST	9251S
SAPK/JNK	Rabbit IgG	CST	9252S
β-catenin (D10A8)	Rabbit IgG	CST	8480S
GAPGH	Rabbit IgG	Proteintech	10494-1-AP
β-actin	Rabbit IgG	Proteintech	20536-1-AP

Supplementary Table 7. Primers for PCR-based genotyping analysis.

Gene	Forward primer (5′–3′)	Reverse primer (5′–3′)
GAPGH	ACCACAGTCCATGCCATCAC	TCCACCACCCTGTTGCTGTA
Luc (luciferase)	AGATACGCCCTGGTTCCTGG	ACGAACACCACGGTAGGCTG
Cre	GAACCTGATGGACATGTTCAGG	AGTGCGTTCGAACGCTAGAGCCTGT
